# Relationship of device measured physical activity type and posture with cardiometabolic health markers: pooled dose–response associations from the Prospective Physical Activity, Sitting and Sleep Consortium

**DOI:** 10.1007/s00125-024-06090-y

**Published:** 2024-03-13

**Authors:** Matthew N. Ahmadi, Joanna M. Blodgett, Andrew J. Atkin, Hsiu-Wen Chan, Borja del Pozo Cruz, Kristin Suorsa, Esmee A. Bakker, Richard M. Pulsford, Gregore I. Mielke, Peter J. Johansson, Pasan Hettiarachchi, Dick H. J. Thijssen, Sari Stenholm, Gita D. Mishra, Armando Teixeira-Pinot, Vegar Rangul, Lauren B. Sherar, Ulf Ekelund, Alun D. Hughes, I.-Min Lee, ProPASS collaboration, Andreas Holtermann, Annemarie Koster, Mark Hamer, Emmanuel Stamatakis

**Affiliations:** 1 Mackenzie Wearables Research Hub, Charles Perkins Centre, The University of Sydney, Sydney, NSW Australia; 2 School of Health Sciences, Faculty of Medicine and Health, The University of Sydney, Sydney, NSW Australia; 3 Institute of Sport, Exercise and Health, Division of Surgery and Interventional Sciences, UCL, London, UK; 4 School of Health Sciences and Norwich Epidemiology Centre, University of East Anglia, Norwich, UK; 5 School of Public Health, The University of Queensland, Brisbane, QLD Australia; 6 Department of Sports Science and Clinical Biomechanics, University of Southern Denmark, Odense, Denmark; 7 Biomedical Research and Innovation Institute of Cádiz (INiBICA) Research Unit, University of Cádiz, Cádiz, Spain; 8 Faculty of Education, University of Cádiz, Cádiz, Spain; 9 Department of Public Health, University of Turku and Turku University Hospital, Turku, Finland; 10 Centre for Population Health Research, University of Turku and Turku University Hospital, Turku, Finland; 11 Department of Medical BioSciences, Radboud University Medical Center, Nijmegen, the Netherlands; 12 Department of Physical Education and Sports, Faculty of Sport Sciences, Sport and Health University Research Institute (iMUDS), University of Granada, Granada, Spain; 13 Faculty of Health and Life Sciences, University of Exeter, Exeter, UK; 14 Occupational and Environmental Medicine, Department of Medical Sciences, Uppsala University, Uppsala, Sweden; 15 Occupational and Environmental Medicine, Uppsala University Hospital, Uppsala, Sweden; 16 School of Public Health, Faculty of Medicine and Health, The University of Sydney, Sydney, NSW Australia; 17 HUNT Research Centre, Department of Public Health and Nursing, Faculty of Medicine and Health Sciences, Norwegian University of Science and Technology (NTNU), Trondheim, Norway; 18 School of Sport, Exercise and Health Sciences, Loughborough University, Loughborough, UK; 19 Department of Sport Medicine, Norwegian School of Sport Sciences, Oslo, Norway; 20 Department of Chronic Diseases, Norwegian Public Health Institute, Oslo, Norway; 21 MRC Unit for Lifelong Health and Ageing, UCL Institute of Cardiovascular Science, UCL, London, UK; 22 UCL BHF Research Accelerator, University College London, London, UK; 23 University College London Hospitals NIHR Biomedical Research Centre, London, UK; 24 Division of Preventive Medicine, Brigham and Women’s Hospital and Harvard Medical School, Boston, MA USA; 25 Department of Epidemiology, Harvard T.H. Chan School of Public Health, Boston, MA USA; 26 National Research Centre for the Working Environment, Copenhagen, Denmark; 27 Department of Social Medicine, CAPHRI Care and Public Health Research Institute, Maastricht University, Maastricht, the Netherlands

**Keywords:** Cardiometabolic health, Individual participant meta-analysis, Physical activity type, Posture, Running, Sitting, Stair climbing, Standing, Walking, Wearables

## Abstract

**Aims/hypothesis:**

The aim of this study was to examine the dose–response associations of device-measured physical activity types and postures (sitting and standing time) with cardiometabolic health.

**Methods:**

We conducted an individual participant harmonised meta-analysis of 12,095 adults (mean ± SD age 54.5±9.6 years; female participants 54.8%) from six cohorts with thigh-worn accelerometry data from the Prospective Physical Activity, Sitting and Sleep (ProPASS) Consortium. Associations of daily walking, stair climbing, running, standing and sitting time with a composite cardiometabolic health score (based on standardised *z* scores) and individual cardiometabolic markers (BMI, waist circumference, triglycerides, HDL-cholesterol, HbA_1c_ and total cholesterol) were examined cross-sectionally using generalised linear modelling and cubic splines.

**Results:**

We observed more favourable composite cardiometabolic health (i.e. *z* score <0) with approximately 64 min/day walking (*z* score [95% CI] −0.14 [−0.25, −0.02]) and 5 min/day stair climbing (−0.14 [−0.24, −0.03]). We observed an equivalent magnitude of association at 2.6 h/day standing. Any amount of running was associated with better composite cardiometabolic health. We did not observe an upper limit to the magnitude of the dose–response associations for any activity type or standing. There was an inverse dose–response association between sitting time and composite cardiometabolic health that became markedly less favourable when daily durations exceeded 12.1 h/day. Associations for sitting time were no longer significant after excluding participants with prevalent CVD or medication use. The dose–response pattern was generally consistent between activity and posture types and individual cardiometabolic health markers.

**Conclusions/interpretation:**

In this first activity type-specific analysis of device-based physical activity, ~64 min/day of walking and ~5.0 min/day of stair climbing were associated with a favourable cardiometabolic risk profile. The deleterious associations of sitting time were fully attenuated after exclusion of participants with prevalent CVD and medication use. Our findings on cardiometabolic health and durations of different activities of daily living and posture may guide future interventions involving lifestyle modification.

**Graphical Abstract:**

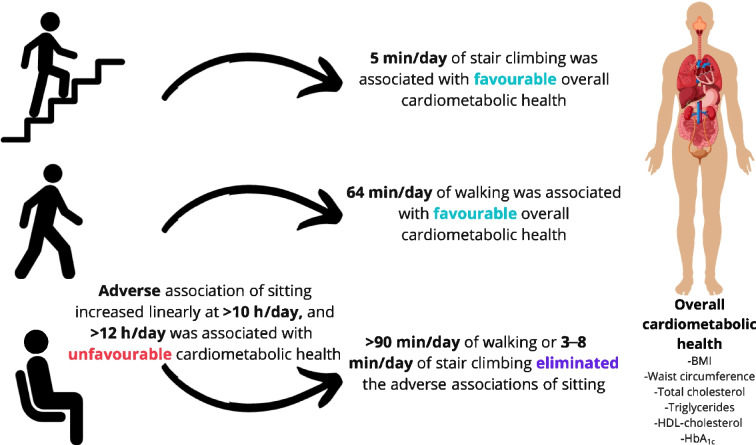

**Supplementary Information:**

The online version contains peer-reviewed but unedited supplementary material available at 10.1007/s00125-024-06090-y.


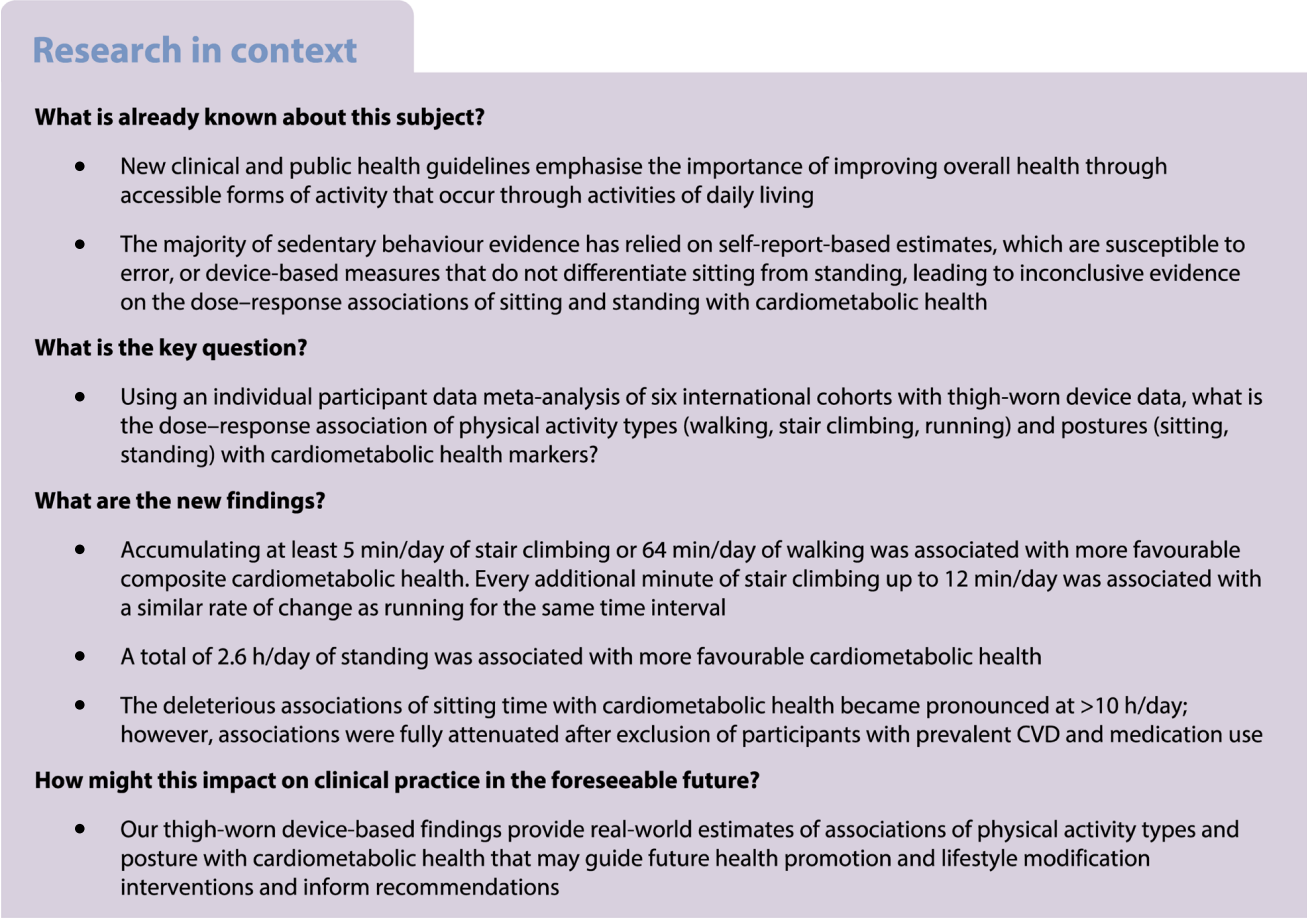


## Introduction

Cardiometabolic risk factors tend to cluster through abnormal metabolic, lipid and non-lipid profiles, leading to increased risk of the development and progression of CVD. It is estimated that more than a quarter of the world’s population will have impaired glucose tolerance by 2045, with 10.9% diagnosed with diabetes [1]. Currently, more than a third of the population is living with hypertension [2], approximately a quarter are classified as overweight, and an additional 13% are classified as obese [3, 4]. Low physical activity and high sedentary time are leading behavioural risk factors [5, 6] for cardiometabolic diseases, but there is a dearth of information on the dose–response relationships between daily time spent in different physical activity types and postures (sitting, standing) and key cardiometabolic outcomes. The latest American Heart Association [6] and European Society of Cardiology [5] reports have identified the need to improve physical activity prescription through accessible forms of daily activity. Similarly, the 2020 WHO Guidelines Development Group highlighted the paucity of evidence on the dose–response relationship of physical activity types with health outcomes and emphasised the value of device-based measurement [7] captured in free-living environments.

Research on the health effects of physical activity has predominantly focused on amounts of intensity-specific physical activity, usually measured through questionnaires. Self-reported physical activity measures are limited as they capture only continuous physical activity blocks lasting a minimum of 10–15 min, are unable to accurately measure posture (e.g. standing time) and are susceptible to recall and social desirability bias [8]. Previous device-based methods relied on acceleration magnitude cut-points to classify activity by intensity, but these cannot determine activity type or posture (e.g. sitting vs standing). Studies using advanced device data curation techniques, which are able to quantify movement and posture at a very high resolution, have identified ‘micropatterns’ of physical activity that are associated with lower mortality [9, 10] and disease incidence [11, 12] risk. Although these wrist device-based outcomes are a significant advance over previous evidence, these studies are limited in assessing associations of posture and physical activity types, including activities of daily living such as stair climbing and running, with health outcomes. Thigh-worn accelerometry, in addition to measuring ambulatory activity type, can differentiate between sitting and standing postures using the tilt angle of the thigh with a high degree of accuracy and consistency [13, 14]. Cross-sectional, single-cohort analyses have shown associations of physical activity or posture with cardiometabolic health markers, although there have been mixed findings for HbA_1c_ [15–17], which is an important marker for diabetes risk and prognosis, in addition to risk of CHD and stroke [18, 19]. Individual participant data meta-analyses (harmonisation of individual participant data from multiple cohorts into a single dataset [20]) of thigh-worn accelerometry and cardiometabolic health markers may improve precision and statistical power, and the generalisability of findings, and possibly provide further clarity to previously inconclusive research. Interventions, using thigh-worn accelerometers, have shown that increased standing time and reductions in sitting time can improve cardiometabolic health outcomes under structured and controlled conditions [21–23]. However, the translatability of these interventions to real-world environments and comparability to ambulatory activity types remain largely unknown.

Using data from the largest pooled thigh-worn accelerometry resource currently available, we conducted a harmonised individual participant data meta-analysis of six cohorts to examine the cross-sectional dose–response associations of device-measured physical activity types (walking, stair climbing, running) and postures (sitting, standing) with cardiometabolic health markers.

## Methods

### Studies

**Fig. 1:**
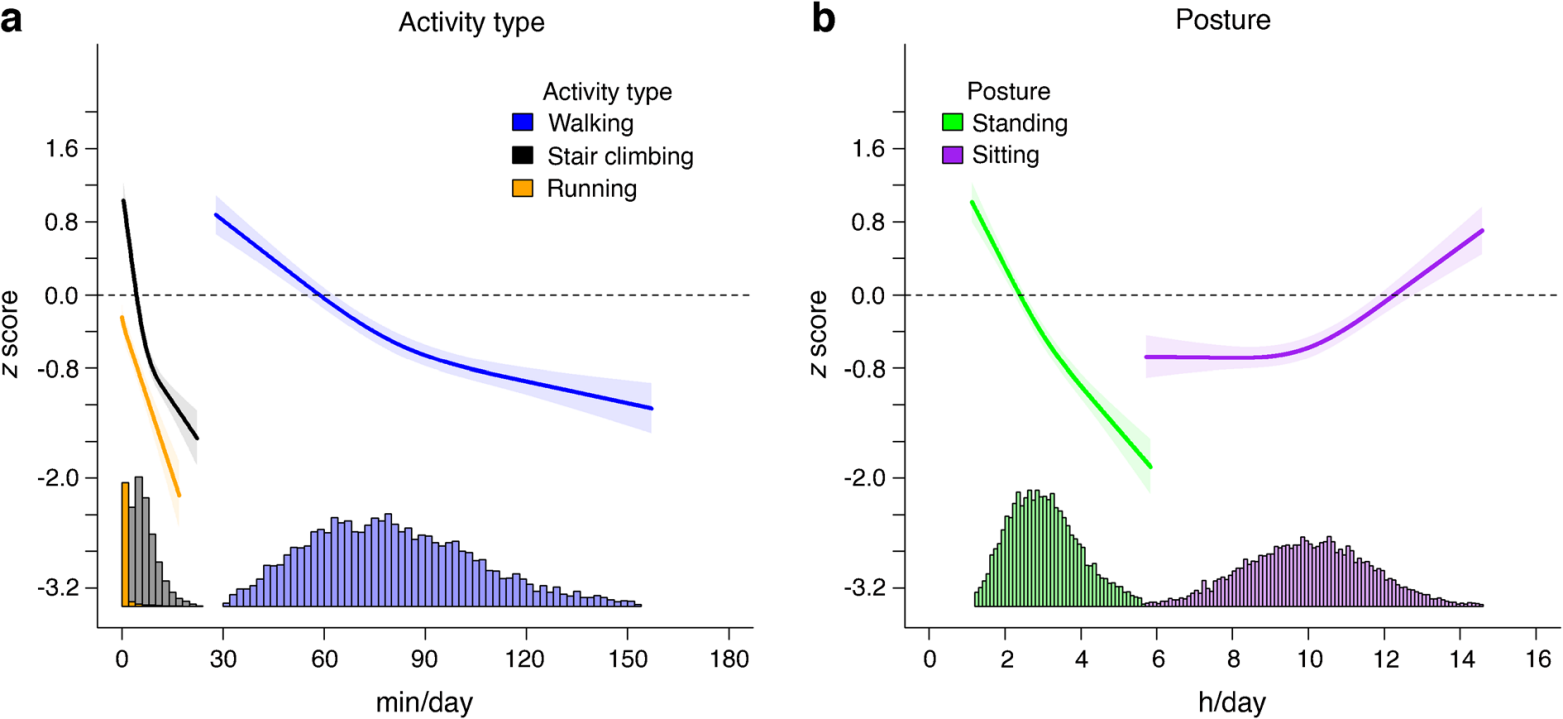
Association of physical activity types (**a**) and posture (**b**) with overall cardiometabolic health. Adjusted for age, sex, smoking, alcohol consumption, sleep duration, self-rated health, medication use, prevalent CVD and cohort, and mutually adjusted for physical activity types and posture using the residual method. *n*=9001. Data shown are point estimates (95% CI). The horizontal dotted line indicates a *z* score of 0. Histograms represent the time distribution for each activity type and posture. Covariate effect size estimates are shown in ESM Table 4

The Prospective Physical Activity, Sitting and Sleep (ProPASS) Consortium is a data resource and research methods development platform that brings together existing and future observational studies of device-measured movement behaviours [24, 25]. The current analyses included pooled individual participant data from six of the ProPASS population cohorts with available cardiometabolic outcomes: the Australian Longitudinal Study on Women’s Health (ALSWH) [26, 27], 1970 British Cohort Study (BCS70) [28], Danish Physical Activity Cohort (DPhacto) [29], Finnish Retirement and Aging Study (FIREA) [30], Nijmegen Exercise Study [31] and The Maastricht Study [32]. In total, 15,168 participants had ≥1 day of valid accelerometer data [33] (≥20 h of wear time and ≥3 h of sleep). We excluded participants with missing covariate data or missing outcomes (electronic supplementary material [ESM] Fig. 1). When collected, participant ethnicity was self-reported. Participant sex and gender were not considered as part of the study design. The study findings are generalisable to the sexes included in the study population.

### Harmonisation of physical activity type and posture

Participants in each cohort were instructed to wear a tri-axial accelerometer capturing raw signal data on their thigh for 24 h a day for 7 days. All accelerometry data cleaning, processing and harmonisation was conducted at the University of Sydney. To ensure consistency in data cleaning and standardisation in processing of accelerometer data, we used a specialised and validated software (ActiPASS v1.32) [34]. ActiPASS autocorrects for device orientation and uses standard procedures for device calibration and identification of non-wear time [35, 36]. Physical activity and posture were classified in 2 s windows with a 50% overlap (resolution of 1 s windows) using a decision tree (Acti4) [37]. The ALSWH, BCS70, Nijmegen Exercise Study and Maastricht Study used ActivPAL monitors (sampling frequency 20 Hz); FIREA used Axivity monitors (sampling frequency 100 Hz); and DPhacto used ActiGraph monitors (sampling frequency 30 Hz).The decision tree model has been shown to have good to excellent accuracy (>90% for sitting, walking and running) for activity type and posture predictions between different monitors [13, 14]. A complete description of the decision tree physical activity type and posture classifier and independent validation are provided in ESM Methods. The signal SD and tilt angle were used to classify fundamental activities and postures such as walking, stair climbing, running, sitting and standing [37]. Sleep was classified using a second decision tree [38]. Mean daily time spent in each activity type and posture was derived by dividing the total duration for individual activity types and postures by the total number of valid wear days for each participant.

### Cardiometabolic health

During clinic or home visits, staff from each cohort recorded participants’ height, weight and waist circumference using standard procedures. Participants from all cohorts except DPhacto provided blood samples for measurement of HDL-cholesterol, total cholesterol, triglycerides and HbA_1c_. Blood biomarker assessment procedures and assay coefficients of variations by cohort are provided in ESM Table 1.

Standardised values (*z* scores based on composite sample distribution) for normalised cardiometabolic markers were calculated [39]. A composite cardiometabolic health score was calculated as the mean of the normally distributed six standardised scores. For HDL-cholesterol, values were inverted, as higher HDL-cholesterol levels are protective for CVD [40]. Sex-specific waist circumference scores were generated to align with sex-specific guidelines [41]. A *z* score of 1 indicates a score of 1 SD above the mean (*z*=0) of the sample, and lower composite scores represent better cardiometabolic health.

### Covariates

For each participating cohort, covariates were measured during clinic or home visits and chosen a priori based on previous literature indicating that they were likely confounders [33, 42, 43] These were age (years), sex (male/female), smoking status (non-smoker/current smoker), alcohol consumption (cohort-specific tertiles based on weekly consumption), self-rated health (5 point Likert scale), self-reported medication use (blood pressure, glucose and lipid-lowering medications), self-reported history of CVD, and cohort. Fasting status was included as a covariate for analyses that included blood biomarker outcomes. Accelerometer-measured sleep duration (hours/day) was also included as a covariate. Daily duration of physical activity types, standing and sitting were mutually adjusted for each other using the residual method [44], consistent with previous studies assessing physical activity over a fixed time interval. For example, in analyses with walking as the exposure, total duration of physical activity was regressed on walking time with the residuals of total physical activity duration used as covariates in our model. A subset of cohorts provided information on highest attained education (*n*=4 cohorts; high school, further education, university/college education or higher), occupational class (*n*=5 cohorts; not working, low occupational class, intermediate occupational class, high occupational class) and functional mobility (*n*=4 cohorts; ten item questionnaire scores ranging from 0 [lowest] to 100 [highest]). Covariate harmonisation procedures are provided in ESM Table 2.

### Analyses

We conducted a one-stage individual participant data meta-analysis [20] using generalised linear regression to estimate the association of the exposures with compositive cardiometabolic health, BMI, waist circumference, HDL-cholesterol, triglycerides, HbA_1c_ and total cholesterol. Data are presented as beta coefficients with 95% CIs. Assumptions for regression analyses were checked using residuals and leverage vs residual squared plots. To account for potential non-linearity of the association between physical activity types (walking, running, stairs) and postures (sitting, standing) and each outcome, we used restricted cubic spline modelling with knots at the 10th, 50th and 90th percentiles. Departure from linearity was assessed using a Wald test, examining the null hypothesis that the coefficient of the second spline was equal to 0.

In sensitivity analyses of composite cardiometabolic health, for participants with available data (i.e. ALSWH, BCS70 and The Maastricht Study), we included adjustments for socioeconomic status (education and occupational class) and functional mobility. We also repeated our analyses after excluding participants with prevalent CVD (*n*=1162) or medication use (blood pressure, glucose or lipid-lowering medications; *n*=3360). To assess if the associations of sitting time with cardiometabolic health varied by daily duration of different activity types, we performed a stratified analysis by grouping walking and stair climbing into low, medium and high categories. To assess the influence of missing data, we included an analysis of composite cardiometabolic health using multiple imputation by chained equations for missing covariate data [45]. We tested for interactions (ANOVA) between each exposure sex. If an interaction was significant, we performed additional analyses stratified by sex. To account for associations that might be due to differences in the absolute time spent in different physical activity types and postures, we performed an analysis for composite cardiometabolic health with time standardised (*z* score) for each exposure.

We performed all analyses using R statistical software (version 4.3.1; Vienna, Austria) with the rms package (version 6.7.0). We report this study in accordance with the Preferred Reporting Items for Systematic Reviews and Meta-Analyses of individual participant data (PRISMA IPD checklist; see ESM).

## Results

### Participant characteristics

Our analytical sample included 12,095 participants. Descriptions of the individual cardiometabolic markers and participant characteristics by cohort are provided in Table 1. Mean age was 54.5 years (SD 9.6), 54.8% of participants were female and 43.5% had very good to excellent self-rated health. Participants in the Nijmegen Exercise Study cohort had the highest observed stair climbing time (median [IQR] 9.5 [6.3, 14.9] min/day) and participants in DPhacto had the highest walking time (98.1 [79.8, 121.8] min/day). Collectively, participants from the FIREA, Nijmegen Exercise Study and Maastricht Study cohorts had the highest sitting time, at a median of >10 h/day. The characteristics of the excluded participants are shown in ESM Table 3.

**Table 1:** Participant characteristics by cohort (*n*=12,095)

Characteristic	ALSWH	BCS70	DPhacto	FIREA	Nijmegen Exercise Study	The Maastricht Study	Overall
Sample	870	3782	290	221	121	6811	12,095
Age, years	44.6 (1.8)	46.8 (0.7)	47.5 (9.5)	62.9 (1.0)	65.6 (7.7)	59.9 (8.7)	54.5 (9.6)
Female, *n* (%)	870 (100.0)	1927 (51.0)	146 (50.3)	181 (81.9)	50 (41.3)	3460 (50.8)	6634 (54.8)
Sedentary time, h/day (median [IQR])	9.6 (8.4, 10.6)	9.0 (7.9, 10.2)	9.1 (7.8, 10.8)	10.1 (8.6, 11.1)	10.3 (9.5, 11.2)	10.2 (9.1, 11.3)	9.8 (8.5, 11.0)
Standing time, h/day (median [IQR])	3.3 (2.6, 4.1)	2.8 (2.2, 3.5)	3.8 (3.0, 4.6)	3.2 (2.6, 4.1)	2.5 (2.0, 3.1)	3.0 (2.3, 3.7)	3.0 (2.3, 3.7)
Walking time, min/day (median [IQR])	84.6 (68.2, 102.7)	71.3 (55.3, 89.2)	98.1 (79.8, 121.8)	82.8 (67.7, 100.0)	90.0 (71.1, 110.7)	79.2 (61.7, 98.8)	77.7 (60.4, 97.2)
Stair climbing time, min/day (median [IQR])	4.9 (2.9, 7.7)	6.4 (4.2, 9.6)	5.9 (3.8, 9.2)	6.8 (4.0, 10.5)	9.5 (6.3, 14.9)	6.1 (3.9, 8.9)	6.1 (3.9, 9.1)
Running time, min/day (median [IQR])	0.4 (0.2, 1.0)	0.3 (0.1, 0.7)	0.3 (0.1, 0.6)	0.2 (0.1, 0.6)	0.4 (0.1, 4.8)	0.2 (0.1, 0.5)	0.2 (0.1, 0.6)
Sleep, h/day	8.2 (1.1)	6.2 (1.0)	7.2 (1.2)	7.4 (1.0)	7.6 (0.9)	7.8 (1.2)	7.3 (1.3)
Valid wear days (median [IQR])	6 (6, 6)	6 (6, 6)	4 (3, 5)	4 (3, 4)	6 (6, 6)	7 (6, 7)	6 (6, 7)
Current smoker, *n* (%)	54 (6.2)	635 (16.8)	86 (29.7)	11 (5.0)	3 (2.5)	834 (12.2)	1623 (13.4)
Self-rated health, *n* (%)							
Excellent	143 (16.4)	809 (21.4)	15 (5.2)	106 (48.0)	16 (13.2)	381 (5.6)	1470 (12.2)
Very good	403 (46.3)	1482 (39.2)	169 (58.3)	79 (35.7)	91 (75.2)	1561 (22.9)	3785 (31.3)
Good	255 (29.3)	1023 (27.0)	98 (33.8)	31 (14.0)	12 (9.9)	3982 (58.5)	5401 (44.7)
Fair	57 (6.6)	374 (9.9)	7 (2.4)	4 (1.8)	2 (1.7)	837 (12.3)	1281 (10.6)
Poor	12 (1.4)	94 (2.5)	1 (0.3)	1 (0.5)	0 (0.0)	50 (0.7)	158 (1.3)
Alcohol consumption, *n* (%)							
Tertile 1 (lowest)	262 (30.1)	1368 (36.2)	87 (30.0)	73 (33.0)	43 (35.5)	2258 (33.2)	4091 (33.8)
Tertile 2	341 (39.2)	1301 (34.4)	102 (35.2)	75 (33.9)	38 (31.4)	2271 (33.3)	4128 (34.1)
Tertile 3 (highest)	267 (30.7)	1113 (29.4)	101 (34.8)	73 (33.0)	40 (33.1)	2282 (33.5)	3876 (32.0)
Medication use^a^, *n* (%)	50 (5.7)	369 (9.8)	92 (31.7)	9 (4.1)	73 (60.3)	3167 (46.5)	3760 (31.1)
Prevalent CVD, *n* (%)	21 (2.4)	100 (2.6)	6 (2.1)	11 (5.0)	20 (16.5)	1116 (16.4)	1274 (10.5)
Cardiometabolic markers							
BMI, kg/m^2^	27.8 (6.4)	27.0 (5.1)	28.1 (5.0)	26.5 (4.6)	25.5 (3.3)	26.8 (4.4)	27.0 (4.8)
Waist circumference, cm							
Men	–	99.3 (11.7)	98.8 (11.7)	101.0 (11.7)	–	100.7 (11.8)	100.1 (11.8)
Women	89.2 (14.9)	88.2 (13.0)	93.5 (13.4)	89.8 (12.3)	–	89.1 (12.5)	88.9 (13.0)
Total cholesterol, mmol/l	3.6 (0.9)	3.9 (1.1)	–	3.9 (0.9)	3.5 (1.0)	3.6 (1.1)	5.3 (1.1)
HDL-cholesterol, mmol/l	1.6 (0.4)	1.6 (0.4)	–	1.8 (0.5)	1.5 (0.4)	1.6 (0.5)	1.6 (0.5)
Triglycerides, mmol/l	1.3 (0.9)	1.8 (1.3)	–	1.2 (0.5)	1.3 (1.1)	1.4 (0.9)	1.5 (1.0)
HbA_1c_							
mmol/mol	32.7 (4.1)	36.0 (6.4)	–	–	–	39.0 (9.2)	37.9 (8.4)
%	5.14 (2.53)	5.44 (2.74)	–	–	–	5.72 (2.99)	5.62 (2.92)

Data are mean (SD) unless noted otherwise

^a^Lipid-modifying, hypertensive and glucose-lowering medications

### Multivariable adjusted dose–response associations of activity type and posture with a composite cardiometabolic health score

Running and stair climbing had the strongest relationship with cardiometabolic health in terms of activity duration and association magnitude (Fig. 1a). For example, any duration of running and ~5 min/day of stair climbing were associated with more favourable cardiometabolic health (i.e. *z* score <0; ~5 min/day of stair climbing *z* score [95% CI] −0.14 [−0.24, −0.03]). When stair climbing exceeded 5.0 min/day, every additional minute up to 12 min/day was associated with a mean *z* score change of −0.09 [−0.10, −0.08]. For the same time interval, every additional minute of running was associated with a *z* score change of −0.11 (−0.13, −0.09). Walking 64 min/day was associated with more favourable cardiometabolic health and a *z* score of −0.14 (−0.25, −0.02). The dose–response association gradient of walking and cardiometabolic health became less steep after 113 min/day of walking (e.g. *z* score change of <0.01 for every additional minute of walking). In comparison, a minimum of 2.6 h/day (156 min/day) of standing (*z* score −0.14 [−0.25, −0.03]) was required to observe more favourable cardiometabolic health (Fig. 1b). For sitting time, the dose–response association became more pronounced at greater than 10 h/day, with greater than 12.1 h/day sitting time associated with an unfavourable cardiometabolic profile (i.e. *z* score >0; Fig. 1b).

### Multivariable adjusted dose–response associations of activity type and posture with individual cardiometabolic health markers

#### Adiposity markers

**Fig. 2:**
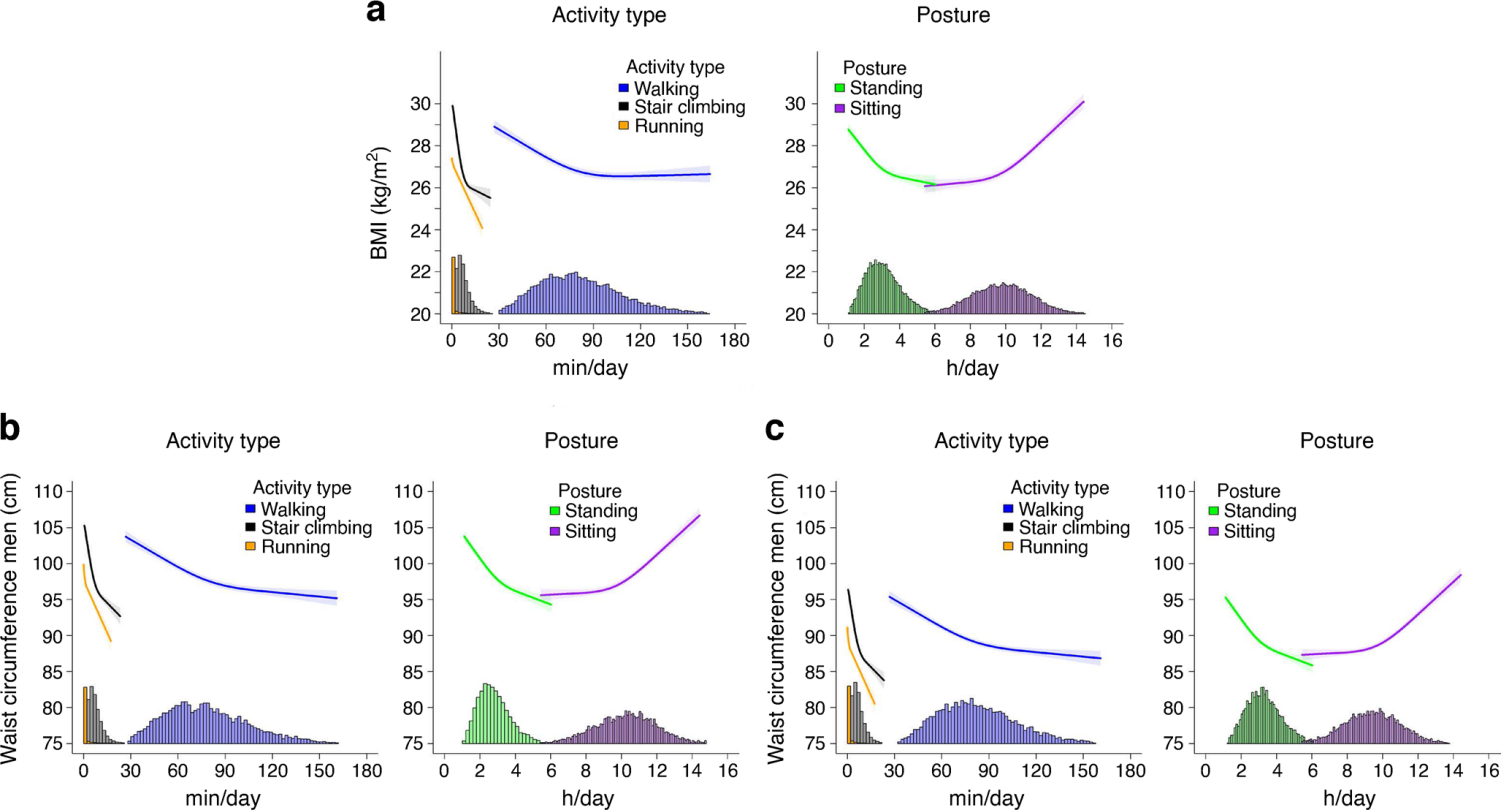
Association of physical activity types and posture with BMI (**a**) and waist circumference in men (**b**) and women (**c**). Adjusted for age, sex, smoking, alcohol consumption, sleep duration, self-rated health, medication use, prevalent CVD and cohort, and mutually adjusted for physical activity types and posture using the residual method. BMI, *n*=12,095; waist circumference, *n*=11,897. Data shown are point estimates (95% CI). Histograms represent the time distribution for each activity type and posture

We observed an inverse dose–response association of standing, walking, stair climbing and running with BMI, although the magnitude of association differed across time for these physical activity types and posture (Fig. 2a). For example, 2.9 (95% CI 2.7, 3.1) h/day of standing, 72.4 (67.8, 78.2) min/day of walking, 6.1 (5.7, 6.6) min/day of stair climbing and 1.2 (0.8, 2.0) min/day of running were associated with a BMI of 27.0 kg/m^2^ (sample mean). The dose–response association for standing, walking and stair climbing began to level off at approximately 3.5 h/day, 90 min/day and 10 min/day, respectively. Higher sitting time was associated with higher BMI, with changes in the magnitude of association becoming pronounced between 9.5 and 10.5 h/day. These association patterns were similar for waist circumference stratified by sex (Fig. 2b,c). For both men and women, the dose–response association for standing, walking and stair climbing levelled off at approximately 3.2 h/day, 90 min/day and 10 min/day, respectively.

#### Biomarkers

**Fig. 3:**
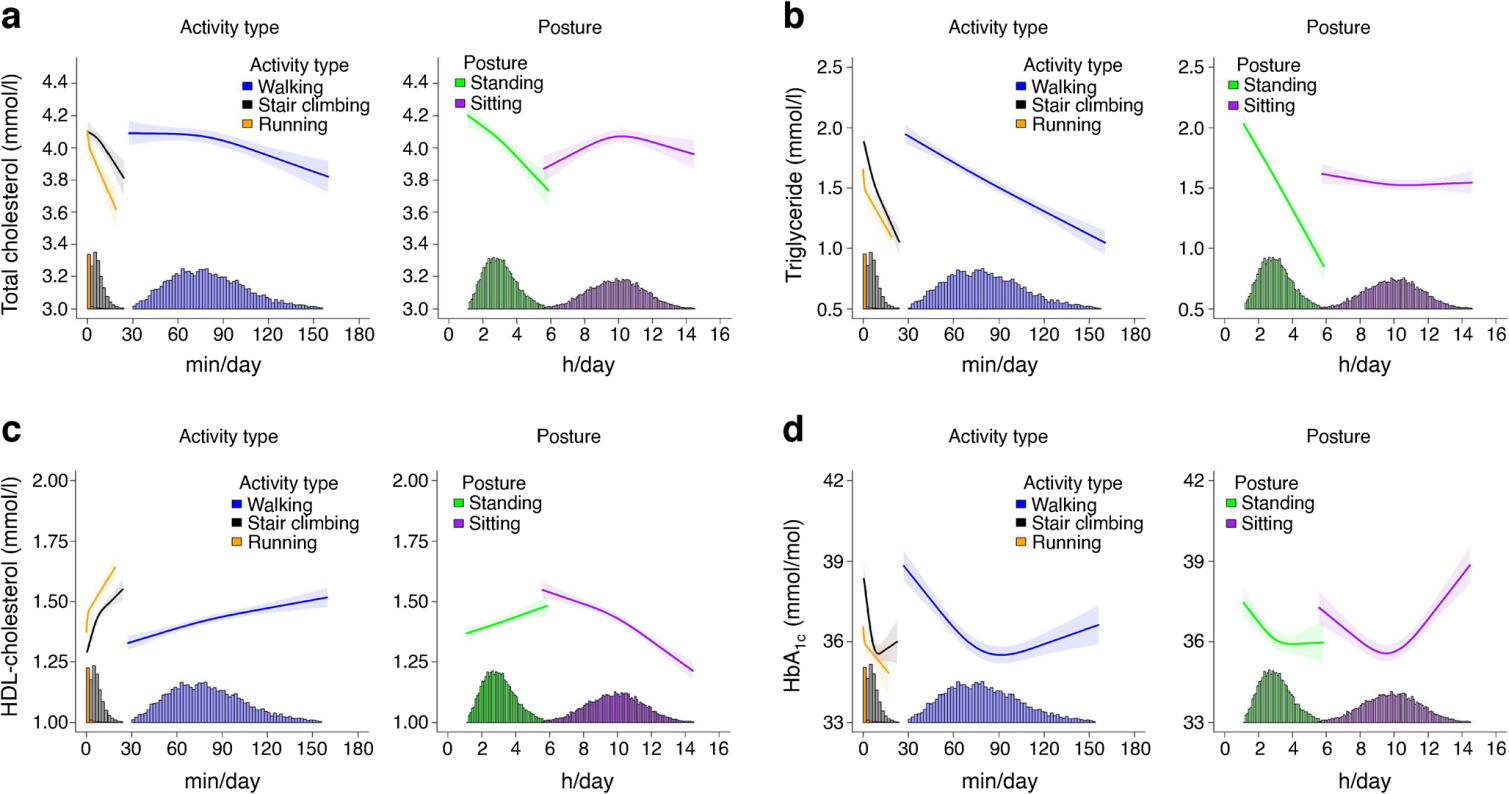
Association of physical activity types and posture with total cholesterol (**a**), triglyceride (**b**), HDL-cholesterol (**c**) and HbA_1c_ (**d**). Adjusted for age, sex, smoking, alcohol consumption, sleep duration, self-rated health, medication use, prevalent CVD and cohort, and mutually adjusted for physical activity types and posture using the residual method. Total cholesterol, *n*=10,728; triglycerides, *n*=9417; HDL-cholesterol, *n*=10,729; HbA_1c_, *n*=10,346. Data shown are point estimates (95% CI). Histograms represent the time distribution for each activity type and posture

We observed an inverse association of time spent in each activity type and standing with total cholesterol (Fig. 3a). There was a stronger magnitude of association for stair climbing and running for a given total cholesterol level. For example, 3.5 (95% CI 3.1, 3.9) h/day of standing, 105.4 (91.2, 121.6) min/day of walking, 11.3 (8.3, 14.9) min/day of stair climbing and 1.4 (0.6, 3.8) min/day of running were associated with a total cholesterol level of 3.9 mmol/l (indicative of low CVD risk [46, 47]). The magnitude of associations for stair climbing and running were nearly parallel for activity levels between 2 min/day and 12 min/day, with about a 0.17 mmol/l difference in total cholesterol (e.g. 4% difference). We observed a linear association between total cholesterol and sitting time up to 10.4 (10.1, 10.7) h/day.

For every additional minute of stair climbing or running, triglyceride levels were lower by a mean of −0.04 (−0.05, −0.03) mmol/l, but with a stronger magnitude of association for running at a given time duration (Fig. 3b). In comparison, every additional 5 min of walking and 10 min of standing were associated with a mean −0.03 (−0.04, −0.02) mmol/l lower triglyceride level. This association pattern with standing, walking, stair climbing and running was similar for HDL-cholesterol (Fig. 3c). Throughout the sitting time duration, there were no significant variations in triglyceride level, but there was an inverse linear association for HDL-cholesterol.

We observed an inverse near linear association between HbA_1c_ and running (Fig. 3d). For stair climbing and walking, the nadir of the dose–response curve was at approximately 10.3 min/day (associated with 35.6 [35.2, 35.9] mmol/mol HbA_1c_) and 91.4 min/day (associated with 35.5 [35.2, 35.8] mmol/mol HbA_1c_), respectively, after which there was a diminishing protective association. A similar association pattern was observed for standing time, with the nadir at 4.1 h/day (associated with 35.9 [35.6, 36.3] mmol/mol HbA_1c_). We observed a J-shaped association between HbA_1c_ and sitting time, with incrementally higher HbA_1c_ levels when daily sitting time exceeded 10.7 h/day.

### Additional and sensitivity analyses

We observed sex interactions for the associations of stair climbing, running and sitting time with the composite cardiometabolic health score (ESM Figs 2–4). There was a stronger protective association for women at any given time duration after approximately 3.9 min/day of stair climbing and 12 s/day of running. For sitting time, we observed the interaction at 10 h/day, after which there was a lower composite cardiometabolic health score (e.g. steeper *z* score curve) for women for higher sitting times.

Association patterns across activity types and posture with composite cardiometabolic health did not change after adjustment for (1) socioeconomic status (occupation and highest attained education level) and (2) functional mobility (ESM Fig. 5). Composite cardiometabolic health results were consistent after standardising the distributions for time spent in each activity type and posture (ESM Fig. 6). After exclusion of participants with prevalent CVD or medication use, we observed inverse linear associations between each activity type and standing and composite cardiometabolic health (ESM Fig. 7), whereas associations for sitting time were fully attenuated. After stratification by walking duration we found that the association of sitting time with composite cardiometabolic health was effectively eliminated in the highest daily walking duration group (≥90 min/day), while there was also evidence of attenuation in the medium daily walking duration group (>60 and <90 min/day) (ESM Fig. 8). We made similar observations in the analyses stratified by stair climbing. For example, in the medium and high stair climbing groups (>3 min/day), there was very little evidence of a dose–response between sitting and composite cardiometabolic health (ESM Fig. 9). The associations between physical activity type and posture and composite cardiometabolic health using multiple imputation for missing covariate data were broadly consistent with the main analysis (ESM Fig. 10).

## Discussion

To our knowledge, this is the first large-scale analysis of type-specific physical activity and posture time, using the first pooled harmonised resource of thigh-worn accelerometry. The placement of accelerometers on the thigh allowed us to accurately derive a range of activity types and postures using novel classification methods to examine their associations with cardiometabolic health markers. Time spent in physical activity types—walking, stair climbing and running—was associated with composite and individual cardiometabolic health markers following adjustment for sitting time and other relevant confounding factors. Accumulating at least 5 min/day of stair climbing, 64 min/day of walking or any duration of running was associated with more favourable composite cardiometabolic health, whereas 2.6 h/day of standing showed associations of comparable magnitude. In contrast, the deleterious association of sitting time with adverse cardiometabolic health became pronounced when daily durations exceeded 10 h/day, although the association was no longer significant after exclusion of participants with prevalent CVD and medication use.

We found a similar association rate of change across various cardiometabolic health markers with stair climbing and running when daily durations were <12 min. The dose–response associations that we observed are plausible. Previous RCTs have found that submaximal activities such as stair climbing that are of low vigorous intensity (e.g. 6.0–8.8 metabolic equivalents [METs] [48]) led to significant improvements in insulin sensitivity, HDL-cholesterol and cardiorespiratory fitness [49–51]. These changes are likely to be induced primarily by skeletal muscle responses that contribute to improved mitochondrial volume and capillarisation (higher density), which leads to improved perfusion and better peripheral oxygen extraction [52]. This promotes enhanced capacity for substrate oxidation, greater use of lipids and reduced carbohydrate catabolism. Consistent with our cardiometabolic findings, short bouts of stair climbing have also been found to have positive effects on postprandial blood glucose levels [53–55], suggesting that the timing of physical activity may be equally as important as total duration, particularly among at-risk populations or populations with diabetes. The intensity range of stair climbing may also elicit improvements to the cardiovascular system. Specifically, a stair climbing intervention [56] among participants with coronary artery disease found that 1.5–3 sessions/week of approximately 7 min (equivalent to 10.5–21 min/week) improved ${\dot{V}{\text{O}}}_{2{\text{peak}}}$ by 1 MET, which has been reported to be associated with a clinically significant 15% reduction in mortality risk [57].

The associations and daily durations that we observed provide evidence that is consistent with large-scale prospective studies examining hard clinical endpoints such as CVD mortality and incidence [9, 10, 58, 59]. We found that between 60 and 115 min/day of walking had the strongest positive association with each cardiometabolic outcome. Notably, this time duration is broadly consistent with the accumulated time duration in previous meta-analyses of walking interventions and cardiometabolic health indicators [60, 61]. Using device-based measures and pooled individual participant data meta-analysis, we were able to translate findings from controlled intervention settings to real-world environments. Collectively, our walking, stair climbing and running findings are important from a public health and clinical perspective. Promotion of activities that are typically performed during daily living and do not require dedicated time commitments may enhance adherence, as has been previously reported in rehabilitation programmes [62–65].

Our results showed that there is an approximate 13:1 min/day ratio for walking vs stair climbing to observe an equivalent favourable composite cardiometabolic health association. Relative to the opportunities that most people have, walking 64 min/day may be more feasible than 5 min/day of stair climbing. A total of 5 min of stair climbing would equate to approximately 350 steps, assuming a mean climbing pace of 70 steps/min [66]. Walking may be more feasible and potentially safer for certain population subgroups, such as older adults, and people who do not have regular access to multiple flights of stairs. Previous prospective studies using self-report data have reported the health-enhancing benefits of walking [67, 68]. Our pooled individual participant data meta-analysis, leveraging objective device-based measurements, extends these studies to derive direct comparisons of walking with other activities and provides more precise habitual activity dose–response estimates.

At a population level, considering walking to be of moderate intensity, our results are broadly consistent with smaller interventions comparing prolonged and continuous moderate-intensity exercise with short duration high-intensity exercise [69–71]. RCTs have found that moderate-intensity continuous training has similar effects on cardiometabolic markers as high-intensity interval training at a time ratio of 7–15:1 (e.g. 60 min of moderate-intensity exercise to 4 min of high-intensity exercise), possibly linked to the intermittent exposure to changes in metabolism and blood flow increases [72]. Although not directly measured in our current study, it is likely that the majority of stair climbing was in bouts of short duration, and that the health-enhancing benefits we observed from walking were due to continuous walking that elicits cardiorespiratory adaptations. Previous studies comparing the effects of activities of daily living and structured exercise sessions on cardiometabolic markers such as insulin sensitivity and glycaemic control found that structured exercise sessions did not consistently provide additional benefits, with the two activity domains eliciting similar metabolic changes in skeletal muscle when matched for intensity volume [73, 74]. Our population-based findings are among the first to extend the findings from such lifestyle modification interventions. Taken together, these findings may inform future research strategies or provide additional options for clinicians attempting to modify the physical activity behaviours of people with low adherence to exercise-based programmes.

We observed that more time spent standing was associated with favourable composite cardiometabolic health and individual cardiometabolic markers. These results are consistent with intervention trials that reported positive cardiometabolic effects from standing [22, 75, 76]. However, in our study, standing was also the least time-efficient of all the activities. We observed that approximately 2.6 h/day of standing was significantly associated with more favourable composite cardiometabolic health. While standing stimulates musculoskeletal responses that may elicit positive changes in cardiometabolic markers, a previous meta-analysis showed that standing for 2–4 h/day may also increase the risk of musculoskeletal disorders by 31–34% [77]. We observed adverse composite cardiometabolic health when sitting time was higher than 12.1 h/day. In our study, it is probable that the deleterious association of high sitting time with adverse composite cardiometabolic health is an effect of lower cardiorespiratory fitness [78–81]. Analyses have shown that cardiorespiratory fitness is a mediator of sitting (e.g. sedentary) time/physical activity and explains about 78% of the relationship with cardiometabolic health [82, 83]. Notably, after exclusion of participants with prevalent CVD or medication use, the deleterious associations of sitting time with adverse composite cardiometabolic health were no longer significant, although there was still a linear trend towards worse cardiometabolic health. Further, our stratified analyses of sitting time by walking and stair climbing duration showed that the deleterious association was fully attenuated when walking exceeded 90 min/day or stair climbing exceeded 3 min/day. Collectively, these results suggest that the relationship of sitting time with cardiometabolic health is highly dependent on time spent in various types of physical activity.

### Strengths and limitations

To our knowledge, this is the first large-scale pooled analysis that compares the health associations with time spent in type-specific physical activity and postures using device-based data. Device-based measurements are less susceptible to the inherent limitations of self-reported measures of physical activity, such as recall and social desirability bias, and are able to capture incidental physical activity across the day that cannot be measured with self-report data. This allowed us to examine the potential health value of short durations of different types of activities more accurately. This is also the first individual participant data meta-analysis using device placement on the thigh, which has an accuracy of >95% for detecting sitting time. Previous studies using hip or wrist placement and only acceleration magnitude cut-points have higher false positive rates due to an inability to differentiate between sitting and standing [84]. The harmonised individual participant data meta-analyses involve original data from multiple cohorts as a single study, allowing us to maintain physical activity type and posture in their continuous form and providing more robust estimates of the observed associations [20, 85] than traditional meta-analyses restricted to study-level aggregated data. Our study also has some limitations. Our observational cross-sectional design limited inferences of causality, and influences of reverse causation may be present. We did not adjust the biomarker analyses for adiposity markers to avoid the potential for overadjustment due to the causal link between the two markers [86]. Our analyses included a range of confounding variables; however, residual and unmeasured confounding is still possible, which may introduce bias. Finally, because of differences in measurement protocols between cohorts, some harmonised covariates have lower granularity than the original data collection; nevertheless, methodologies were similar between studies and allowed for the pooling of data across the six cohorts.

## Conclusion

Using the largest individual participant data meta-analysis of thigh-worn accelerometry data we found that approximately 64 min/day of walking and 5 min/day of stair climbing were associated with more favourable composite cardiometabolic health. Every additional minute of stair climbing up to 12 min/day was associated with a similar rate of change as running for the same time interval. Our device-based findings provide novel estimates quantifying the associations of physical activity types and posture with cardiometabolic health outcomes that may guide future interventions and inform recommendations. If confirmed in prospective studies and intervention trials, extensions of this work may also inform future wearable device-based risk prediction.

### Supplementary Information

Below is the link to the electronic supplementary material.

Supplementary file1 (PDF 1850 KB)

## Data Availability

All data requests will need to provide a methodologically sound justification and will require approval from the ProPASS Consortium.

## Abbreviations

ALSWH: Australian Longitudinal Study on Women’s Health
BCS70: 1970 British Cohort Study
DPhacto: Danish Physical Activity Cohort
FIREA: Finnish Retirement and Aging Study
MET: Metabolic equivalent
ProPASS: Prospective Physical Activity, Sitting and Sleep
