# Supplementary material for: Relationship of device measured physical activity type and posture with cardiometabolic health markers: pooled dose–response associations from the Prospective Physical Activity, Sitting and Sleep Consortium
Source: Diabetologia. 2024 Mar 13;67(6):1051–65. doi: 10.1007/s00125-024-06090-y (PMC11058050; doi:10.1007/s00125-024-06090-y)
Supplement: Supplementary file 1 — Supplementary file1 (PDF 1850 KB) [file 125_2024_6090_MOESM1_ESM.pdf]

Electronic Supplemental Material (ESM)

Page	Item
1	**ESM Figure 1:** Flow diagram of participants in the study
2	**ESM Figure 2:** Association of stair climbing and overall cardiometabolic health stratified by sex
3	**ESM Figure 3**: Association of running time and overall cardiometabolic health stratified by sex
4	**ESM Figure 4**: Association of sedentary time and overall cardiometabolic health stratified by sex
5	**ESM Figure 5**: Association of physical activity types and posture with overall cardiometabolic health. Additional adjustment for education, occupation, and functional mobility
6	**ESM Figure 6**: Association of physical activity types and posture with overall cardiometabolic health with time standardised
7	**ESM Figure 7**: Association of physical activity type and posture with overall cardiometabolic health. Exclusion of prevalent CVD and medication use
8	**ESM Figure 8**: Association of sitting time with overall cardiometabolic health. Stratified by walking duration
9	**ESM Figure 9**: Association of sitting time with overall cardiometabolic health. Stratified by walking duration
10	**ESM Figure 10:** Association of physical activity types and posture with overall cardiometabolic health; with multiple imputation for missing covariate data
11	**ESM Table 1:** Assessment of blood biomarkers
15	**ESM Table 2:** Assessment and harmonisation procedures of covariates for each participating cohort
23	**ESM Table 3:** Excluded participant characteristics by cohort

**24**	**ESM Table 4:** Covariate effect size estimates for physical activity type and posture with composite cardiometabolic health
**28**	**ESM Methods:** Physical activity type and posture classification
**30**	**PRISMA-IPD Checklist**

ESM Figure 1: Flow diagram of participants in the study

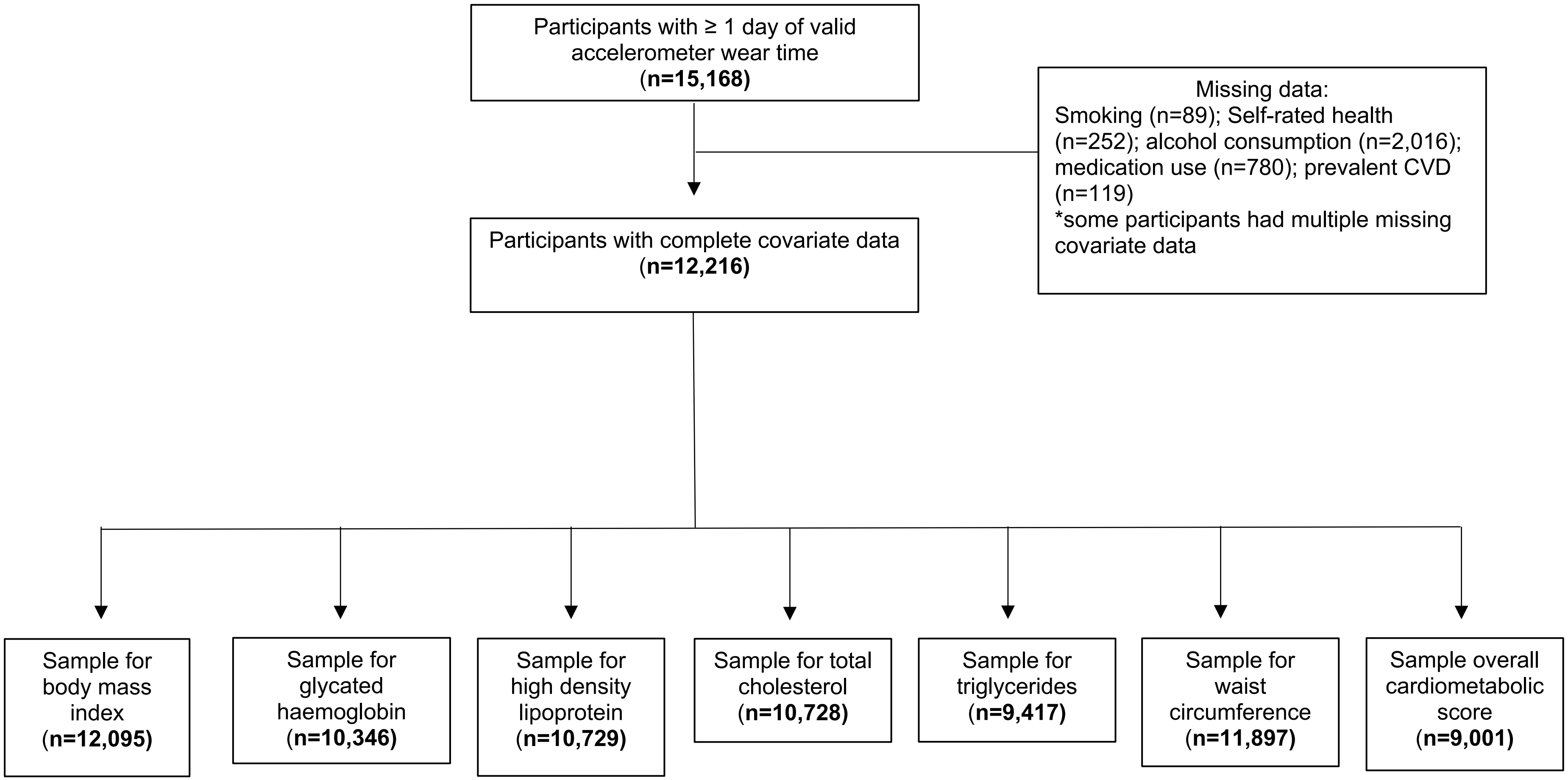

ESM Figure 2: Association of stair climbing and overall cardiometabolic health stratified by sex

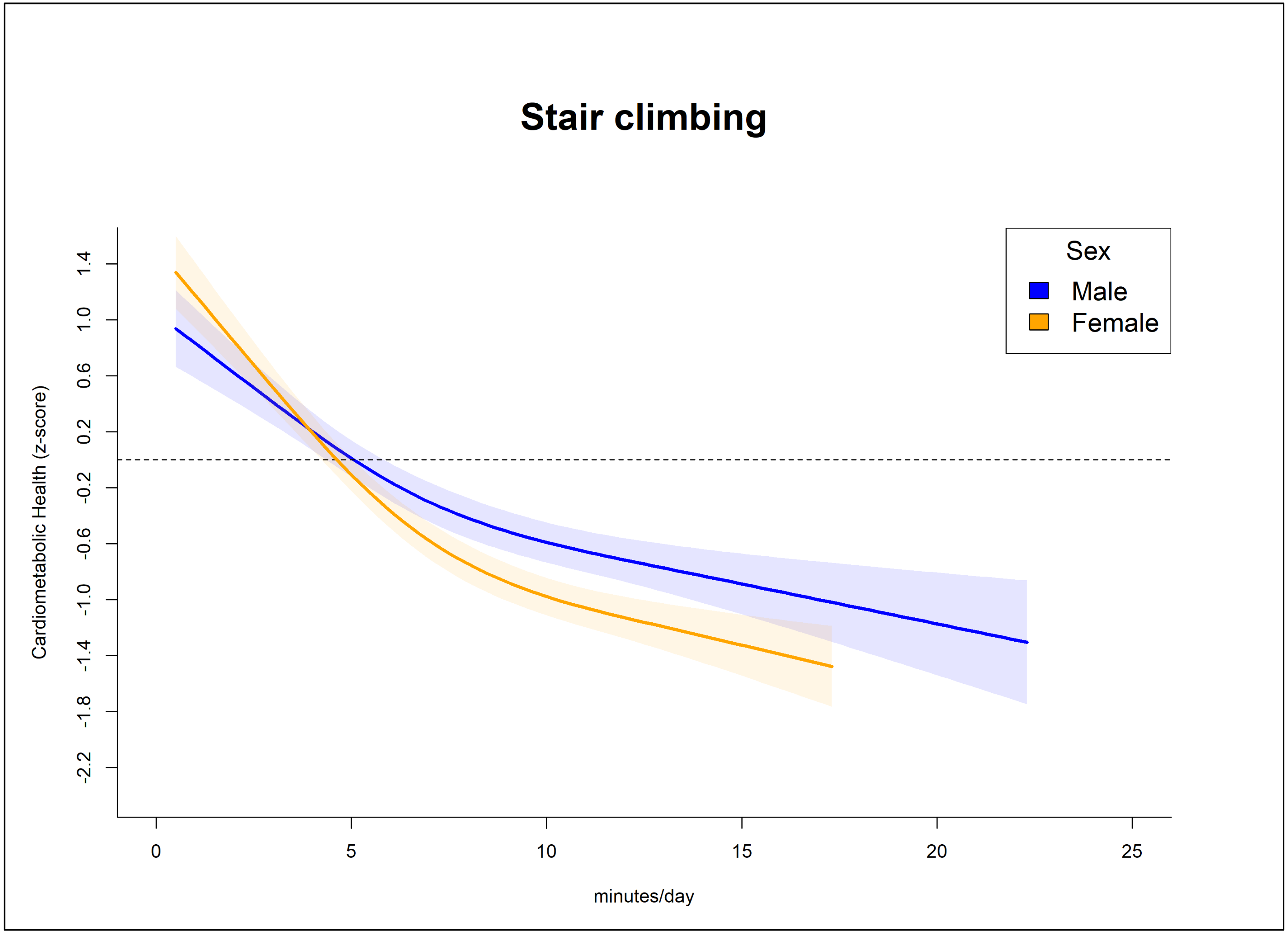

Adjusted for age, sex, smoking, alcohol consumption, sleep duration, self-rated health, medication use, prevalent CVD, cohort, and mutual adjustment for physical activity types and posture using the residual method. N=9,001. Horizontal dotted line indicates a z-score of 0.

ESM Figure 3: Association of running time and overall cardiometabolic health stratified by sex

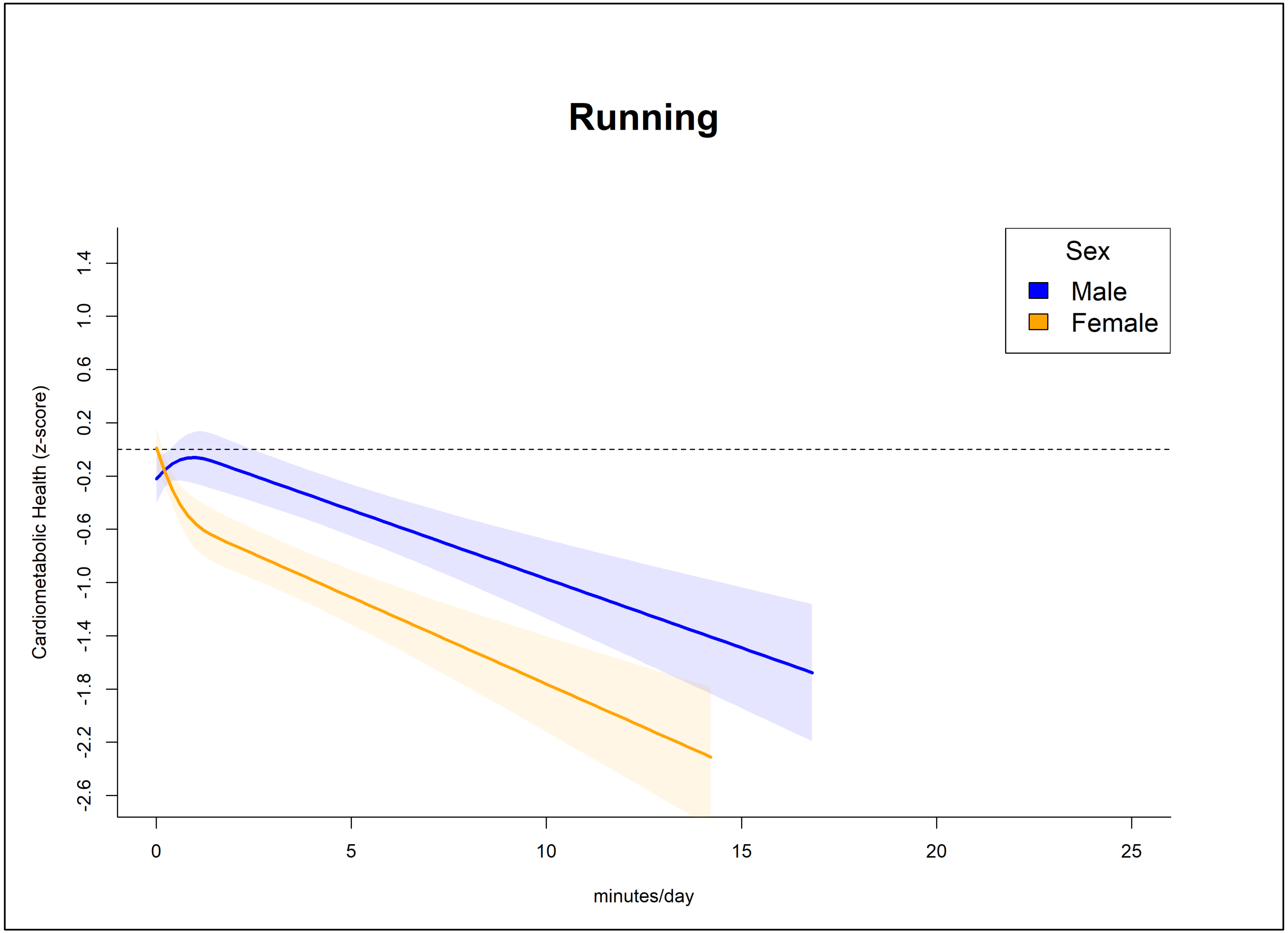

Adjusted for age, sex, smoking, alcohol consumption, sleep duration, self-rated health, medication use, prevalent CVD, cohort, and mutual adjustment for physical activity types and posture using the residual method. N=9,001. Horizontal dotted line indicates a z-score of 0.

ESM Figure 4: Association of sedentary time and overall cardiometabolic health stratified by sex

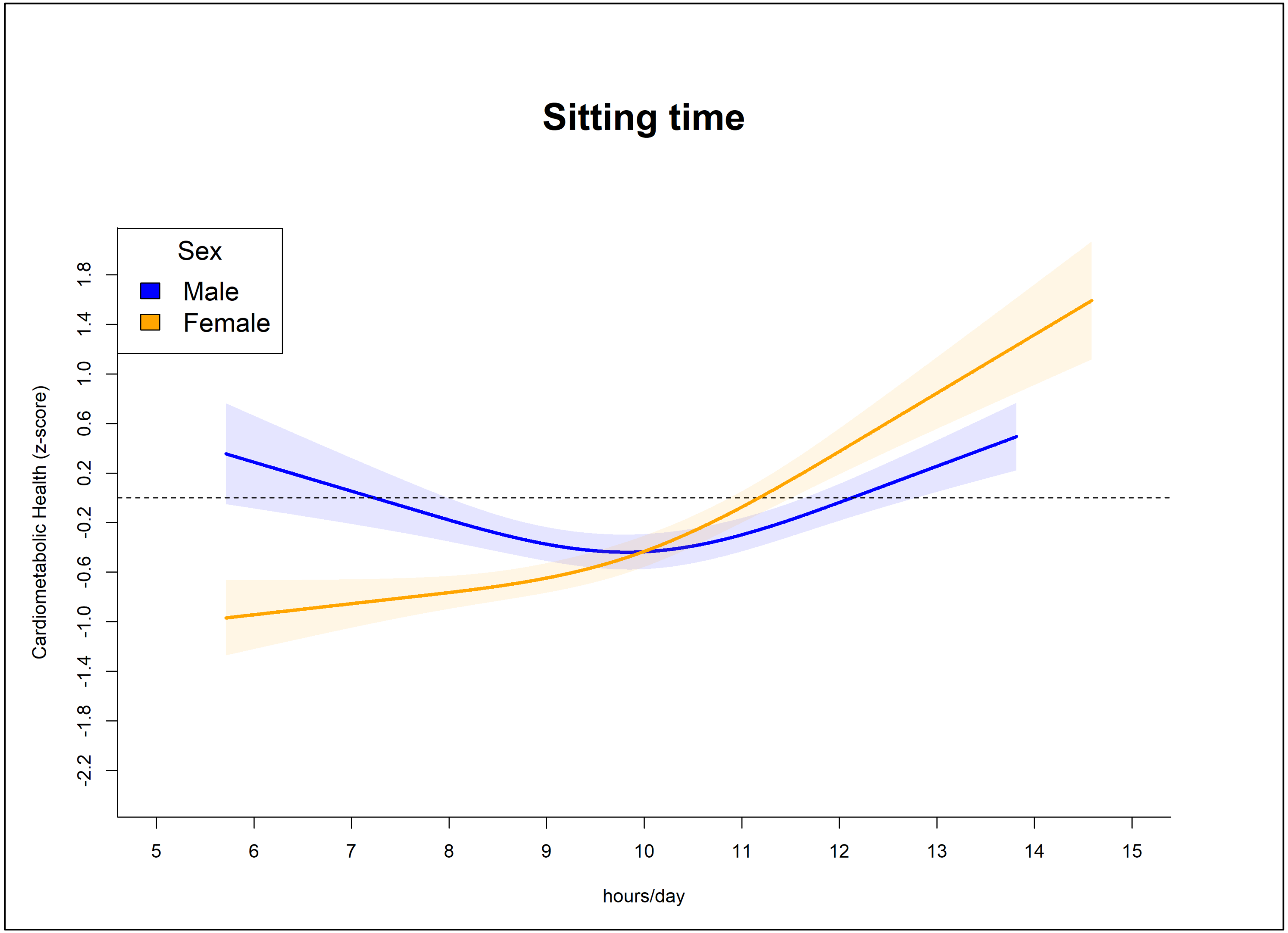

Adjusted for age, sex, smoking, alcohol consumption, sleep duration, self-rated health, medication use, prevalent CVD, cohort, and mutual adjustment for physical activity types and posture using the residual method. N=9,001. Horizontal dotted line indicates a z-score of 0.

ESM Figure 5: Association of physical activity types and posture with overall cardiometabolic health. Additional adjustment for education, occupation, and functional mobility

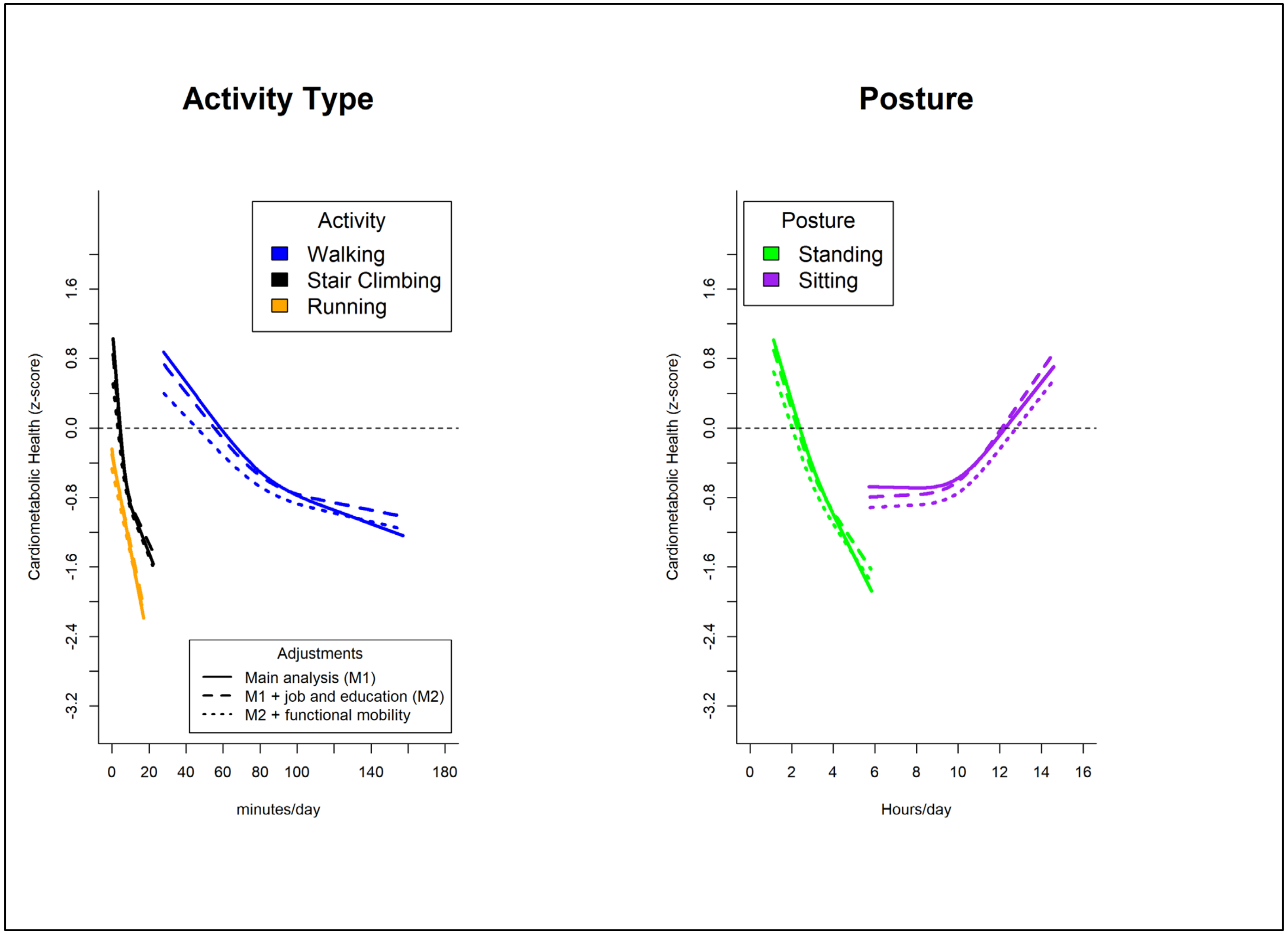

Adjusted for age, sex, smoking, alcohol consumption, sleep duration, self-rated health, medication use, prevalent CVD, cohort, and mutual adjustment for physical activity types and posture using the residual method, job, education, and functional mobility. Main analysis (M1)= 9,013; M1+ job and education (M2)=8,020; M2 + functional mobility=7,978. Horizontal dotted line indicates a z-score of 0

ESM Figure 6: Association of physical activity types and posture with overall cardiometabolic health with time standardised

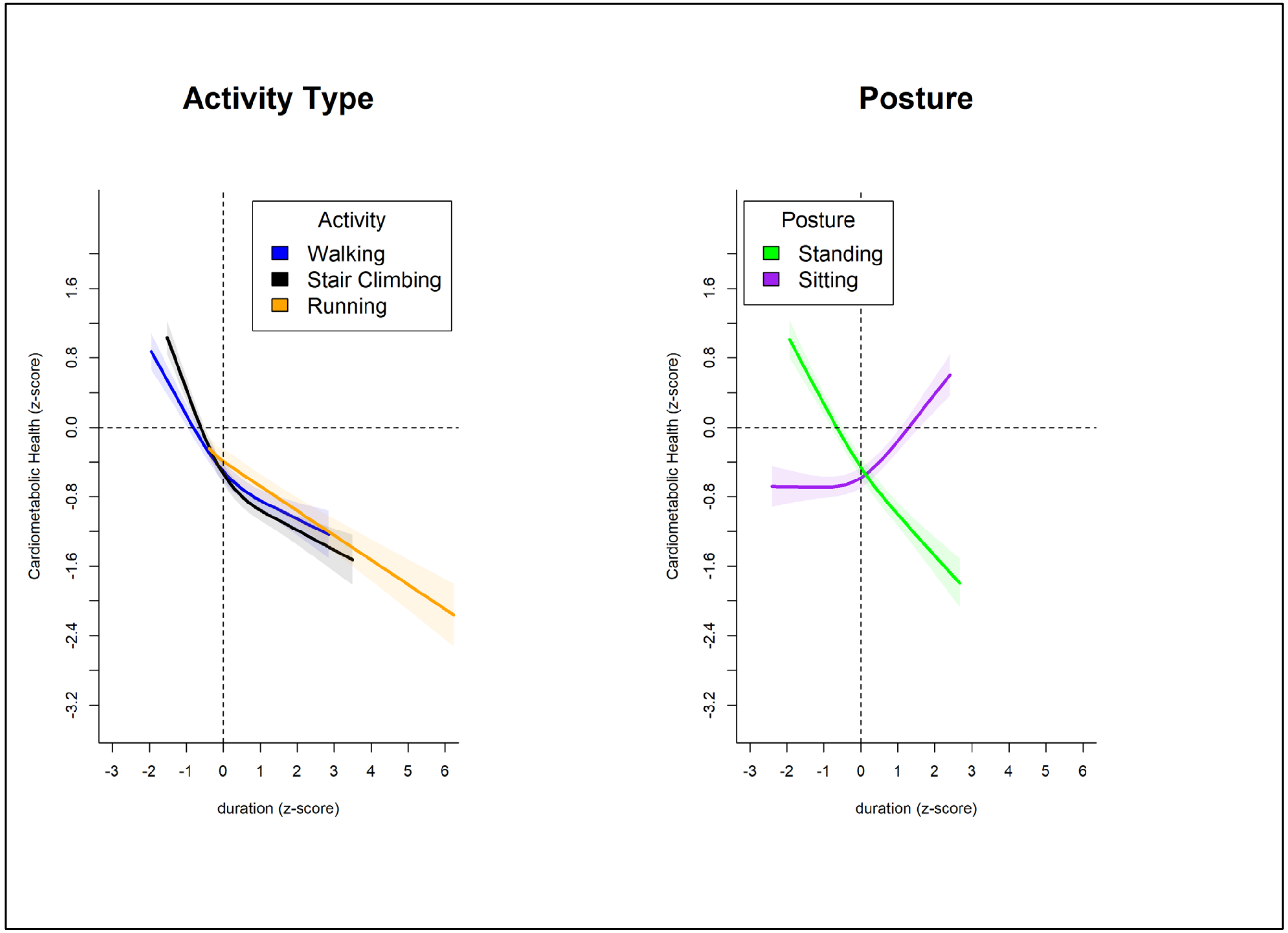

Adjusted for age, sex, smoking, alcohol consumption, sleep duration, self-rated health, medication use, prevalent CVD, cohort, and mutual adjustment for physical activity types and posture using the residual method. N=9,001. Horizontal dotted line indicates a z-score of 0. Vertical dotted line indicates: 7.1 minutes/day of stair climbing, 80.3 minutes/day walking, 1.1 minutes/day running, 3.1 hours/day standing, and 9.8 hours/day sitting.

ESM Figure 7: Association of physical activity type and posture with overall cardiometabolic health. Exclusion of prevalent CVD and medication use

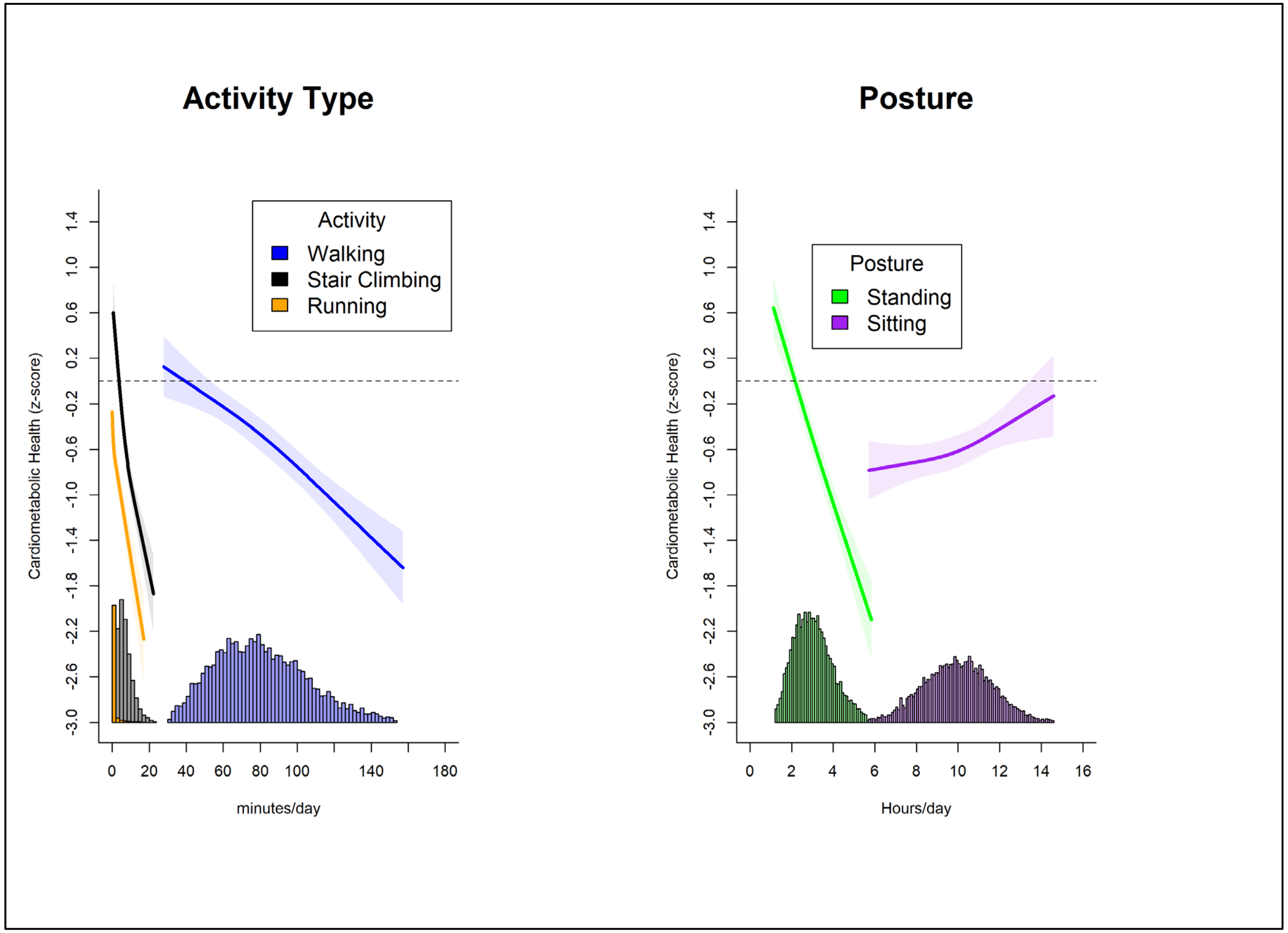

Adjusted for age, sex, smoking, alcohol consumption, sleep duration, self-rated health, cohort, and mutual adjustment for physical activity types and posture using the residual method. N=5,367. Horizontal dotted line indicates a z-score of 0

ESM Figure 8: Association of sitting time with overall cardiometabolic health. Stratified by walking duration

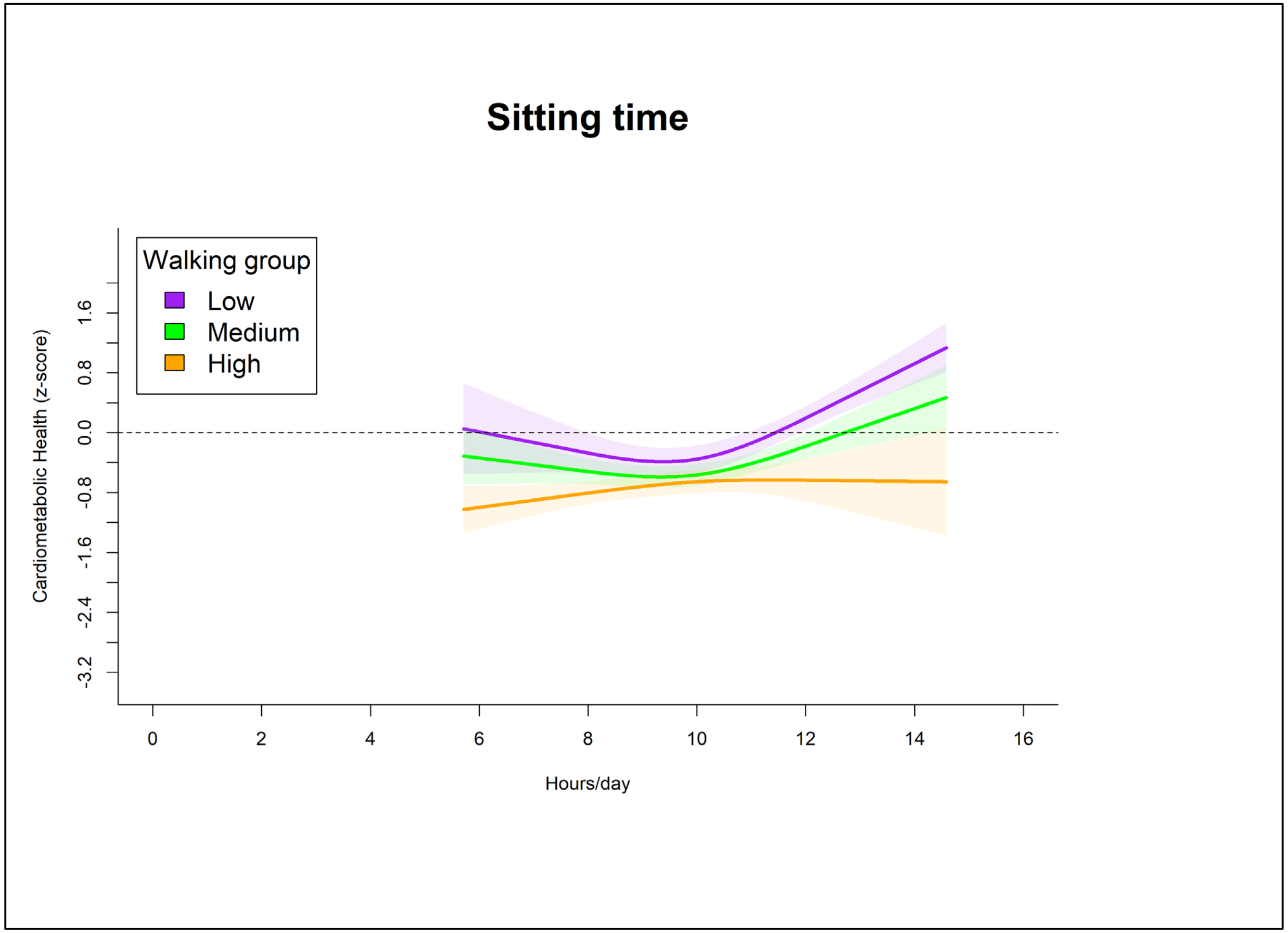

Adjusted for age, sex, smoking, alcohol consumption, sleep duration, self-rated health, cohort, and mutual adjustment for physical activity types and posture using the residual method. Walking group: low= ≤60 mins/day medium= >60 and <90 mins/day; high= ≥90 mins/day. Horizontal dotted line indicates a z-score of 0

ESM Figure 9: Association of sitting time with overall cardiometabolic health. Stratified by stair climbing duration

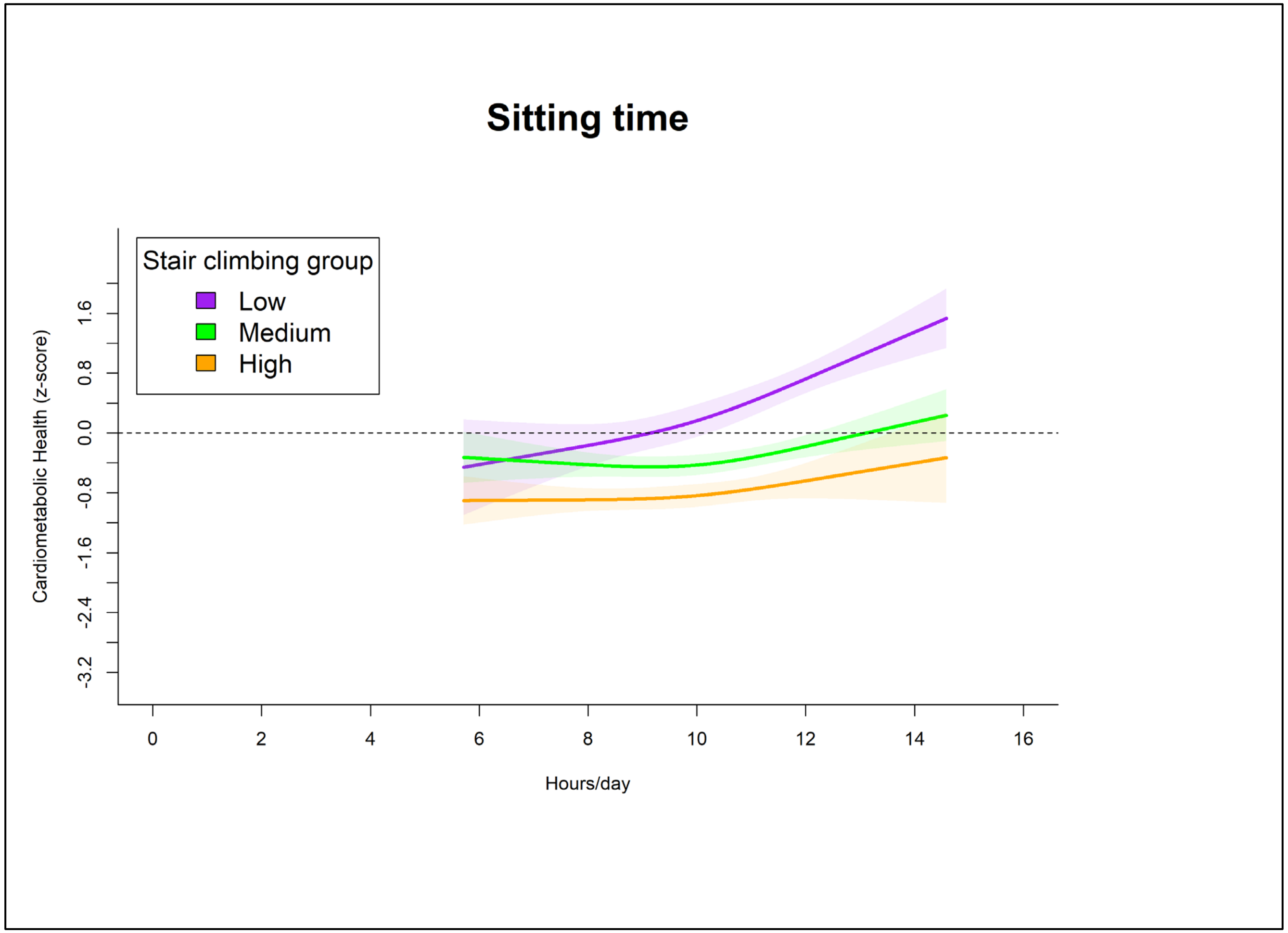

Adjusted for age, sex, smoking, alcohol consumption, sleep duration, self-rated health, cohort, and mutual adjustment for physical activity types and posture using the residual method. Stair climbing group: low= ≤3 mins/day medium= >3 and <8 mins/day; high= ≥8 mins/day. Horizontal dotted line indicates a z-score of 0

ESM Figure 10: Association of physical activity types and posture with overall cardiometabolic health; with multiple imputation for missing covariate data

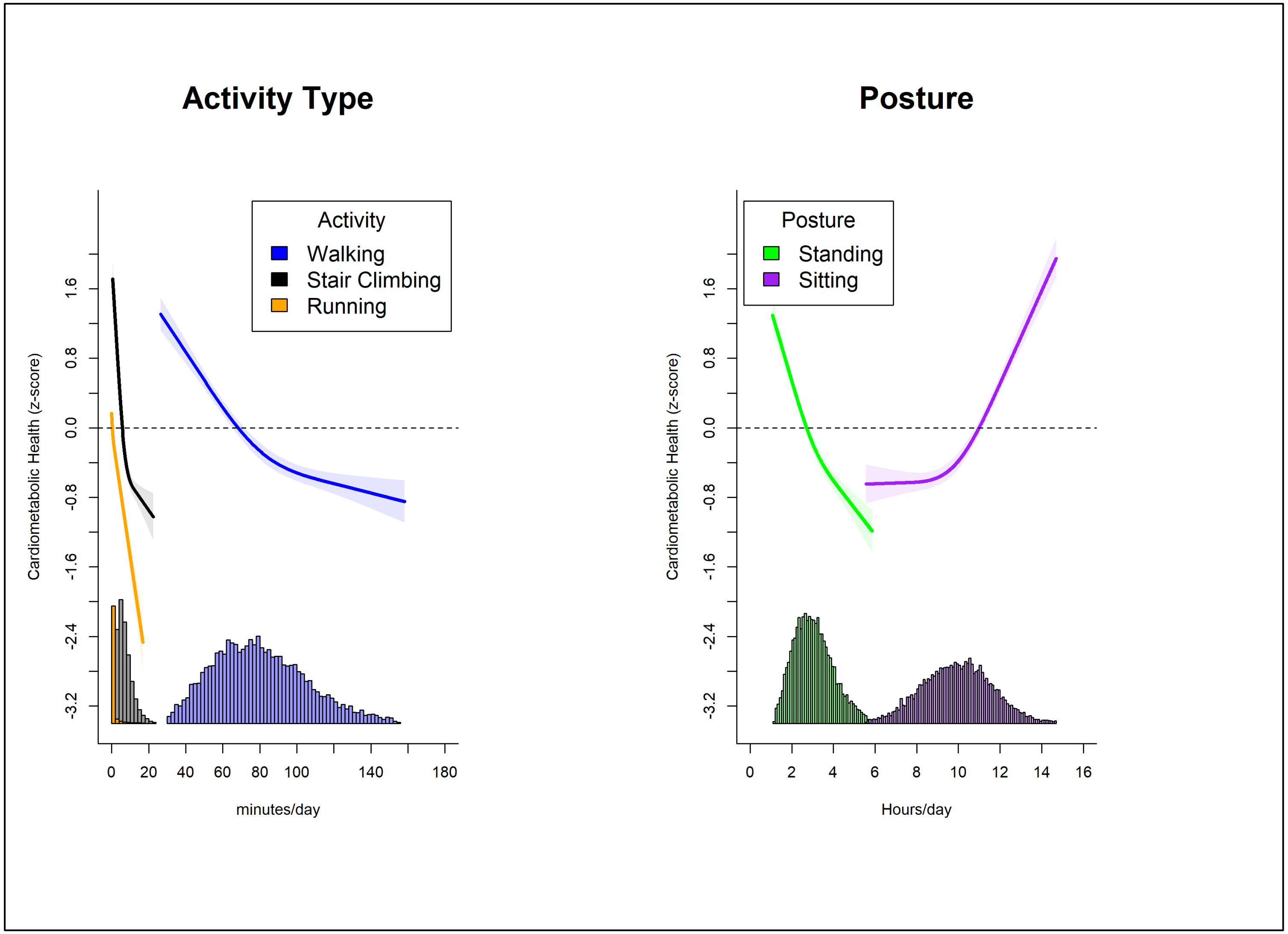

Adjusted for age, sex, smoking, alcohol consumption, sleep duration, self-rated health, medication use, prevalent CVD, cohort and mutual adjustment for physical activity types and posture using the residual method. N=10,397. Horizontal dotted line indicates a z-score of 0.

ESM Table 1: Assessment of blood biomarkers

	Harmonised construct	*Australian Longitudinal Study on Women's Health (n=870)*	*1970 British Birth Cohort Study (n=3,782)*	*Finnish Retirement and Aging Study (n=221)*	*Nijmegen Exercise Study (n=121)*	*The Maastricht Study (n=6,811)*
Collection of blood samples		Nurses collected non-fasting venous blood samples in morning.	Nurses collected non-venous blood samples at various times throughout day.	Nurses collected fasting venous blood samples in the morning.	Blood was draw and freezed (-80 degrees Celcius) and analysed within 5 months.	Trained research staff collected venous blood samples in the morning
Fasted/non-fasted blood sample	0: Non-fasted<br>1: Fasted	0: All participants provided non-fasted sample	0: All participants provided non-fasted sample	1: All participants provided fasted sample.	0: Did not fast for 4 hours OR had light meal 4-8 hours before blood draw<br>1: Fasted for 4 hours AND did not have Light meal 4-8 hours before blood-draw	1: All participants provided fasted sample
Total cholesterol	Continuous measure (mmol/L)	Measured using routine autoanalyser methods.<br><br><u>QLD/VIC samples</u><br>Method principle: Cholesterol oxidase, esterase, peroxidase	Method principle: Enzymatic colourimetric- cholesterol esterase/cholesterol oxidase/peroxidase<br><br>Manufacturer: Roche Cobas	Measured with standard (enzymatic and colorimetric) methods<br><br>Manufacturer: Cobas 8000 c702, Roche Diagnostics<br><br>CV: ≤4.8%	<u>Method priniciple:</u> Enzymatic- cholesterol esterase and cholesterol oxidase<br><br>Manufacturer: Siemens Attelica CH<br><br>CV: ≤1.3%	Measured with standard (enzymatic and/or colorimetric) methods<br><br>Manufacturer: Beckman Synchron LX20, Beckman Coulter Inc., Brea, USA; or Roche Cobas

	Harmonised construct	*Australian Longitudinal Study on Women's Health (n=870)*	*1970 British Birth Cohort Study (n=3,782)*	*Finnish Retirement and Aging Study (n=221)*	*Nijmegen Exercise Study (n=121)*	*The Maastricht Study (n=6,811)*
		Manufacturer: Beckman Coulter DXC800<br>CV: <1.8%<br><br>SA/WA/NSW samples:<br>Method principle: Cholesterol oxidase, esterase, peroxidase<br>Manufacturer: Siemens Atellica<br>CV: <2%	c702, generation 2 assay<br><br>CV: ≤2.6%			6000, Roche diagnostics, Mannheim, Germany<br><br>CV: ≤2.5%
HDL	Continuous measure (mmol/L)	Measured using routine autoanalyser methods.<br><br>QLD/VIC samples<br>Method principle: Direct measure, polymer-polyanion<br><br>Manufacturer: Beckman Coulter DXC800	Method principle: Enzymatic colourimetric-dextran sulphate/PEG-cholesterol esterase/PEG-cholesterol oxidase/peroxidase.<br><br>Manufacturer: Roche Cobas	Measured with standard (enzymatic and colorimetric) methods<br><br>Manufacturer: Cobas 8000 c702, Roche Diagnostics<br><br>CV: ≤6.1%	Method priniciple:<br>Enzymatic-cholesterol esterase and cholesterol oxidase<br><br>Manufacturer: Siemens Attelica CH<br><br>CV: ≤2.0%	Measured with standard (enzymatic and/or colorimetric) methods by an automatic analyzer<br><br>Manufacturer: Beckman Synchron LX20, Beckman Coulter Inc., Brea, USA; or Roche Cobas

	Harmonised construct	Australian Longitudinal Study on Women's Health (n=870)	1970 British Birth Cohort Study (n=3,782)	Finnish Retirement and Aging Study (n=221)	Nijmegen Exercise Study (n=121)	The Maastricht Study (n=6,811)
		CV: <3%<br><br>SA/WA/NSW samples<br>Method principle: Direct measure, polymer-polyanion<br>Manufacturer: Siemens Atellica<br>CV: <3.2%	c702, generation 3 assay<br><br>CV: ≤2.8%			6000, Roche diagnostics, Mannheim, Germany<br><br>CV: ≤4.5%
Total:HDL cholesterol ratio	Continuous measure (mmol/L)	Derived as total cholesterol/HDL cholesterol				
HbA1c	Continuous measure (mmol/L)	Measured using routine autoanalyser methods.<br><br>Method principle: High performance liquid chromatography<br>Manufacturer: Bio-Rad D100<br>CV: <1.5%	Method principle: Ion exchange HPLC.<br>Manufacturer: Tosoh G8<br><br>CV: ≤3.3%	n/a	n/a	Method principle: Ion-exchange high performance liquid chromatography<br><br>Manufacturer: Variant tm II, Bio-Rad, Hercules, California, USA<br><br>CV: ≤1.2%

	Harmonised construct	*Australian Longitudinal Study on Women's Health (n=870)*	*1970 British Birth Cohort Study (n=3,782)*	*Finnish Retirement and Aging Study (n=221)*	*Nijmegen Exercise Study (n=121)*	*The Maastricht Study (n=6,811)*
Triglycerides	Continuous measure (mmol/L)	Measured using routine autoanalyser methods.<br><br>**QLD/VIC samples**<br><br>Method principle: Enzymatic, end point<br>Manufacturer: Beckman Coulter DXC800<br>CV: <3.3%<br><br>**SA/WA/NSW samples**<br>Method principle: Enzymatic, end point<br>Manufacturer: Siemens Atellica<br>CV: <3.4%	Method principle: Enzymatic colourimetric: lipoprotein lipase/glycerol kinase/glycerol phosphate oxidase/peroxidase Manufacturer: Roche Cobas c702<br>CV: ≤2.4%	Measured with standard (enzymatic and colorimetric) methods<br><br>Manufacturer: Cobas 8000 c702, Roche Diagnostics<br><br>CV: ≤5.3%	Method priniciple: Enzymatic-endpoint<br><br>Manufacturer: Siemens Attelica CH<br><br>CV: ≤2.5%	Measured with standard (enzymatic and/or colorimetric) methods by an automatic analyzer (Beckman Synchron LX20, Beckman Coulter Inc., Brea, USA; or Roche Cobas 6000, Roche diagnostics, Mannheim, Germany<br><br>CV: ≤3.5%

CV: coefficient of variation QLD: Queensland VIC: Victoria SA: South Australia WA: West Australia NSW: New South Wales

ESM Table 2: Assessment and harmonisation procedures of covariates for each participating cohort

Construct	Units	*Australian Longitudinal Study on Women's Health (n=870)*	*1970 British Birth Cohort Study (n=3,782)*	Danish Physical ACTivity cohort with Objective measurement (n=290)	*Finnish Retirement and Aging Study (n=221)*	*Nijmegen Exercise Study (n=121)*	*The Maastricht Study (n=6,811)*
Age	Continuous (years)	Question: "What is your age in years?"	All participants assigned age 46 (birth cohort study; year of birth: 1970; year of accelerometer assessment: 2016)	Determined using workers' unique civil registration number based on time between date of birth and date of measurement visit	Derived based on time between date of birth and date of measurement visit	Derived based on time between date of birth and date of measurement visit	Derived based on time between date of birth and date of measurement visit
Sex	1: Male<br>2: Female	2: Female	1: Male<br>2: Female	1: Male<br>2: Female	1: Male<br>2: Female	1: Male<br>2: Female	1: Male<br>2: Female
Smoking	0: Non-smoker<br>1: Smoker	**Question**: "How often do you currently smoke?"<br>**Responses & coding**:<br>0: Not at all<br>1: Daily; At least weekly (but not daily); Less often than weekly	**Question**: "Which of the statements on this card applies to you?"<br>**Responses & coding**:<br>0: I've never smoked cigarettes; I used to	**Question**: "Do you smoke?"<br>**Responses & coding**:<br>0: Never smoked; Formerly smoked<br>1: Daily smoking; Occasionally smoking	**Question**: "Do you currently smoke or have you smoked regularly, i.e. daily or almost daily?"<br>**Responses & coding**:<br>0: No I have never smoked; Yes, previously<br>1: Yes, currently	**Question**: "Do you smoke?"<br>**Responses & coding**:<br>0: No, but I smoked in the past; No, I have never smoked<br>1: Yes	**Question**: "Do you smoke?"<br>**Responses & coding**:<br>0: No I have never smoked; No, I stopped smoking more than 6 months ago; No, I

			smoke but don't at all<br>1: I now smoke occasionally but not daily; I smoke cigarettes every day					stopped less than 6 months ago<br>1: Yes
Medication use	0: No lipid, glucose, or blood pressure medication<br>1: 1 or more of the above	**Protocol:** Participants were asked to bring all medications to the assessment, which were coded using Anatomical Therapeutic Chemical classification<br><br>Anatomical Therapeutic Chemical classification	**Protocol:** Research nurses collected data on all prescription medications which were coded using British National Formulary edition 69 codes<br><br>1: Any of 0212 : Lipid-Regulating Drugs; 0201 – 0207: hypertension related drugs; 0601 Drugs	**Questions**: “Have you in the last three months been taken prescription medication?” “If yes, what kind of medication?”<br><br>**Responses & coding**:<br>1: Antihypertensive<br>0: No medications or other medications	**Protocol:** Research nurses inquired about all prescription medications	**Questions**: “Did you use medication in the past year?” was asked immediately following positive responses to “Which of the following diseases below has been diagnosed by physician?” for 1) hypercholesterolemia; 2) hypertension; 3) diabetes<br><br>**Responses & coding**:<br>1: Yes for any of above<br>0: No	**Protocol:** Participants were asked to bring all medications to the assessment, which were coded using Anatomical Therapeutic Chemical classification

			Used In Diabetes	**Questions:** "Do you take medication for high blood pressure?<br><br>**Responses & coding**:<br>1: Yes<br>2: No			
Fasted/non-fasted blood sample		0: Non-fasted<br>1: Fasted	0: All participants provided non-fasted sample	0: All participants provided non-fasted sample	0: Did not fast for 4 hours OR had light meal 4-8 hours before blood draw<br>1: Fasted for 4 hours AND did not have Light meal 4-8 hours before blood-draw	1: All participants provided fasted sample	1: All participants provided fasted sample.
History of cardiovascular conditions	0: No history of CVD<br>1: History of CVD	**Questions**:<br>Wave 1:"Have you ever been told by a doctor that you have heart disease"<br>Wave 2: "[In the last 4 years] [In more than 4 years ago], have you ever been told by a doctor that you have heart disease?"	**Questions**: "Since last collection wave, have you had any of the health problems listed on this card? [high blood pressure]"	**Question**: "Do you have angina pectoris?"<br><br>**Responses & coding**:<br>0: No<br>1: Yes	**Questions**: "Has a doctor/physician given you a diagnosis of [angina pectoris]/[myocardial infarction]/[stroke]/[hypertension]?"	**Questions**: "Which type of diseases below has been diagnosed by physician? [myocardial infarction]/ [heart failure]/ [stroke]/ [atrial fibrillation]/ [hypertension]"	**Questions**: Rose Questionnaire<br><br>Responses:<br>1: Selected any of myocardial infarction - cerebrovascular infarction and/or hemorrhage -

		Waves 3-8: "In the last 3 years have you been diagnosed or treated for heart disease?"<br>Wave 8: "In the last 3 years have you been diagnosed or treated for hypertension?"<br><br>**Responses & coding**:<br>0: No to all of above<br>1: Yes to any of above	"Since last collection wave, have you had any of the health problems listed on this card? Please include any health problems that had already started before that date. [heart problems]/ [stroke]".<br><br>**Responses & coding**:<br>0: No to all of above<br>1: Yes to any of above		**Responses & coding**:<br>0: No to all of above<br>1: Yes to any of above	**Responses & coding**:<br>0: No to all of above<br>1: Yes to any of above	percutaneous artery angioplasty of the coronary arteries, abdominal arteries, peripheral arteries or carotid artery - vascular surgery on coronary arteries, abdominal arteries, peripheral arteries or carotid artery.<br>0: selected none of the above
<u>Mobility limitations</u>	Continuous score from 0 to 100 of the SF 10-item physical function, where 0 indicates	All cohorts used the SF-36 scale. The 10-items included limitations in: vigorous activities, moderate activities, lifting and carrying groceries, climbing several flights of stairs, climbing one flight of stairs, bending, kneeling or stooping, walking about two kilometers, walking about a half kilometer, in walking about 100 metres, in bathing or dressing. Each item had three possible responses: Yes, limited a lot (0); Yes, limited a little (50); No, not limited at all (100). Mobility limitations score was calculated as the average score across all ten items.					

	poor mobility and 100 indicates no mobility problems.						
Education	**0**: None or lower than high school<br>**1**: High school qualifications (age 16y)<br>**2**: Further education qualifications (age 16-18y)<br>**3**: university degree and higher (18+y)	**Question**: “What is the highest level of qualification you have completed?”<br><br>**Responses & coding**:<br>**0**: No formal qualifications<br>**1**: Year 10 or equivalent<br>**2**: Year 12 or equivalent, Trade/apprenticeship, Certificate/Diploma<br>**3**: University degree, Higher university degree	**Derived variable** of National Vocational Qualifications categories based on self-reported “recognised academic, vocational, clerical, business or commercial qualifications” asked at each wave<br><br>**Responses & coding**:<br>**0:** No academic qualification<br>**1:** GCDS D-E, GCSE A-C, CSES 2-5, Other Scottish			**Question**: “Please select your highest educational qualification from the list below”<br><br>**Responses & coding**:<br>0: lower education (primary school), lower pre-vocational education (low)<br>1: pre-vocational education (moderate), secondary education (moderate)<br>2: middle-level applied education (moderate)	**Question**: “What is your highest completed educational level?”<br><br>**Responses & coding**:<br>0: None, Uncompleted primary educational level, Primary educational level<br>1: Lower vocational education, Intermediate general secondary education

			qualifications, Good O levels Scottish standards<br>**2**: As levels or 1 A level; 2+ Alevels, Scottish higher/6th, diploma<br>**3**: Degree level, Higher degree			3: higher professional education (high), university (high)	2: Intermediate vocational education, Higher general secondary education, Higher vocational education<br>3: University education
Occupational class	0: Not working<br>1: Low occupational class<br>2: Intermediate occupational class<br>3: High occupational class	**Question**: “What is your main occupation now?”<br><br>**Responses & coding**:<br><br>0: No paid job<br>1: Elementary clerical, sales or service worker; Labourer or related worker<br>2: Tradesperson or related worker; Advanced clerical	**Derived variable** of National Statistics Socio-economic Classification (NS-SEC) based on participants “[description] in [their] own words what [they] mainly did in this job]	**Derived variable** pulled from personnel lists of companies:<br><br>**Coding:**<br>1= production worker AND unskilled<br>2=production worker AND skilled<br>2= Administration and office	**Derived variable** from the Register of Pension Institute Keva of International Standard Classification of Occupations (ISCO)<br><br>**Responses & coding**:<br>1: plant and machine operators, and assemblers;	N/a	**Question**: “Which category best fits your current/past job?”<br><br>**Responses & coding**:<br>0: Not working<br>1: Low occupational class<br>2: Intermediate occupational

		or service worker; Intermediate clerical; sales/service worker; Intermediate production or transport worker 3: Manager or administrator; Professional; Associate professional	**Responses & coding**: 0: Never worked and long-term unemployed 1: L10 Lower supervisory occupations; L11 Lower technical occupations; L12 Semi-routine occupations; L13 Routine occupations 2: L5 Lower managerial and administrative ; L6 Higher supervisory occupations; L7 Intermediate occupations; L8 Employers	workers AND skilled or unskilled	elementary occupations 2: clerical support workers; service and sales workers; skilled agricultural, forestry and fishery workers; craft and related trades workers 3: managers; professionals; technicians and associate professionals		class; Self-employed 3: High occupational class, Professionals

			in small organisations; L9 Own account workers<br>3: L1 Employers in large establishments; L2 Higher managerial and administrative; L3 Higher professional occupations; L4 Lower professional and higher technical				

ESM Table 3: Excluded participant characteristics by cohort

	Australian Longitudinal Study on Women's Health	British Birth Cohort Study	Danish Physical Activity Cohort	Finnish Retirement and Aging Study	Nijmegen Exercise Study	The Maastricht Study	Overall
**Sample**	82	1,457	490	33	416	708	3,186
**Age, years**	44.6 (1.6)	46.8 (0.7)	43.8 (9.8)	62.6 (1.4)	60.7 (11.6)	58.1 (9.0)	50.8 (9.6)
**Female, n (%)**	82 (100.0)	872 (59.8)	214 (43.7)	29 (87.9)	194 (46.6)	332 (46.9)	1,723 (54.1)
**Sedentary time, h/day (median [IQR])**	9.6 [8.2, 10.9]	9.0 [7.7, 10.3]	9.0 [7.6, 10.4]	9.2 [8.1, 10.5]	10.2 [9.1, 11.1]	10.3 [8.9, 11.5]	9.5 [8.1, 10.8]
**Standing time, h/day (median [IQR])**	3.3 [2.5, 4.1]	2.8 [2.1, 3.6]	3.8 [3.0, 4.6]	4.3 [3.6, 4.8]	2.5 [2.0, 3.2]	2.9 [2.3, 3.7]	2.9 [2.2, 3.8]
**Walking time, min/day (median [IQR])**	81.4 [63.7, 109.2]	67.5 [49.7, 86.7]	100.7 [79.1, 128.3]	84.3 [63.8, 100.3]	88.7 [69.2, 110.2]	78.9 [60.7, 98.3]	77.5 [58.5, 99.7]
**Stair climbing time, min/day (median [IQR])**	4.7 [2.4, 8.9]	5.5 [3.5, 8.7]	6.7 [4.2, 10.3]	7.9 [5.6, 10.0]	9.8 [6.5, 13.8]	6.0 [4.0, 9.1]	6.3 [4.0, 9.8]
**Running time, min/day (median [IQR])**	0.4 [0.2, 0.8]	0.2 [0.1, 0.5]	0.4 [0.2, 1.0]	0.3 [0.1, 0.5]	0.6 [0.2, 7.2]	0.2 [0.1, 0.5]	0.3 [0.1, 0.6]

**Sleep, h/day**	8.0 (2.0)	6.1 (1.4)	7.2 (1.5)	6.9 (1.7)	7.6 (1.0)	7.6 (1.7)	6.8 (1.6)
**Current smoker, n (%)**	8 (9.9)	315 (21.6)	133 (28.1)	2 (7.7)	13 (3.1)	120 (18.6)	591 (19.1)
**Self-rated health, n (%)**							
Excellent	7 (20.0)	204 (14.0)	51 (10.7)	11 (33.3)	77 (24.0)	30 (4.9)	380 (13.0)
Very good	14 (40.0)	465 (31.9)	300 (63.0)	17 (51.5)	215 (67.0)	119 (19.4)	1,130 (38.5)
Good	10 (28.6)	427 (29.3)	120 (25.2)	4 (12.1)	23 (7.2)	355 (58.0)	939 (32.0)
Fair	4 (11.4)	241 (16.5)	5 (1.1)	0 (0.0)	6 (1.9)	99 (16.2)	355 (12.1)
Poor	0 (0.0)	120 (8.2)	0 (0.0)	1 (3.0)	0 (0.0)	9 (1.5)	130 (4.4)
**Alcohol consumption, n (%)**							
Tertile 1 (lowest)	22 (26.8)	13 (24.1)	130 (29.0)	14 (42.4)	144 (37.6)	58 (42.6)	381 (33.5)
Tertile 2	34 (41.5)	19 (35.2)	167 (37.2)	10 (30.3)	116 (30.3)	45 (33.1)	391 (34.4)

Tertile 3 (highest)	26 (31.7)	22 (40.7)	152 (33.9)	9 (27.3)	123 (32.1)	33 (24.3)	365 (32.1)
**Medication use, n (%)**	7 (8.5)	209 (14.4)	11 (34.4)	3 (9.1)	24 (22.9)	323 (46.3)	577 (24.0)
**Prevalent CVD, n (%)**	1 (1.2)	51 (3.5)	10 (2.1)	0 (0.0)	45 (10.9)	105 (17.1)	212 (6.9)
**Cardiometabolic markers**							
**Body mass index, kg/m²**	28.1 (6.0)	28.2 (6.0)	26.8 (4.7)	25.5 (5.5)	24.4 (3.2)	27.3 (4.8)	27.2 (5.4)
**Waist circumference, cm**							
Males	-	101.6 (13.8)	95.6 (11.2)	99.3 (9.6)	90.9 (9.9)	101.8 (13.0)	100.2 (13.3)
Females	90.0 (14.3)	91.3 (14.7)	87.3 (12.9)	87.1 (12.1)	81.0 (9.2)	90.0 (13.6)	90.2 (14.2)
**Total cholesterol, mmol/l**	5.3 (1.0)	5.3 (1.0)	-	5.7 (0.8)	5.3 (1.0)	5.1 (1.1)	5.2 (1.0)
**Triglycerides, mmol/l**	1.5 (0.9)	1.9 (1.7)	-	1.2 (0.4)	1.1 (0.5)	1.4 (1.0)	1.6 (1.3)
**Glycated haemoglobin; HbA1c**							

mmol/l	34.3 (3.2)	38.1 (10.0)	-	-	-	39.5 (9.9)	38.6 (10.0)
percentage	5.29 (2.44)	5.64 (3.07)	-	-	-	5.76 (3.06)	5.68 (3.07)
**High density lipoprotein, mmol/l**	1.6 (0.4)	1.4 (0.4)	-	1.8 (0.4)	1.6 (0.4)	1.5 (0.5)	1.5 (0.4)

ESM Table 4: Covariate effect size estimates for physical activity type and posture with composite cardiometabolic health

	Exposure				
**Covariate**	**Stairs**	**Walk**	**Run**	**Stand**	**Sit**
Age	0.013 (0.003)	0.016 (0.004)	0.012 (0.004)	0.016 (0.004)	0.016 (0.003)
Sex (ref= female)	0.306 (0.058)	0.408 (0.058)	0.351 (0.058)	0.391 (0.058)	0.403 (0.058)
Current smoker (ref=Yes)	-0.052 (0.081)	-0.020 (0.081)	-0.005 (0.081)	-0.019 (0.081)	-0.023 (0.081)
Alcohol consumption (ref= lowest tertile)	0.143 (0.036)	0.127 (0.036)	0.108 (0.036)	0.122 (0.036)	0.151 (0.036)
Residual of physical activity types and posture	-0.007 (0.001)	-0.009 (0.001)	-0.009 (0.001)	-0.011 (0.001)	-0.009 (0.001)
Sleep duration	-0.006 (0.001)	-0.005 (0.001)	-0.005 (0.001)	-0.005 (0.001)	-0.005 (0.001)
Self-rated health (ref= Excellent)	0.541 (0.0342)	0.586 (0.034)	0.555 (0.035)	0.584 (0.034)	0.583 (0.034)
Medication use (ref= No)	1.019 (0.066)	1.074 (0.066)	1.058 (0.066)	1.072 (0.066)	1.069 (0.066)
Cohort (ref= ALSWH)	0.002 (0.002)	0.003 (0.002)	0.002 (0.002)	0.003 (0.002)	0.003 (0.002)
Prevalent CVD (ref= Yes)	-0.380 (0.085)	-0.330 (0.086)	-0.317 (0.085)	-0.331 (0.086)	-0.326 (0.085)

Values represent effect size (SD)

ESM Methods: Physical activity type and posture classification

A binary decision tree (BDT) was used to classify physical activity type and posture. BDT's use a recursive partitioning technique for classification. The tree begins with a single root node and splits into branches, leading to further nodes until a leaf node is reached. Every non-leaf node is associated with a binary decision which determines which branch to follow. The BDT is built by identifying an acceleration feature value that splits the data into subgroups with the greatest class purity. The splitting continues in each node until the subgroups (e.g. physical activity type and posture) reach a minimum size or until a stop condition is reached. The acceleration standard deviation and tilt angle (derived from the acceleration signal) were used at the root nodes to classify physical activity type and posture.

To assess the accuracy of the BDT classifier, we performed validation testing in an independent sample of 89 participants (Age= 56.7 ± 11.3; 55.2% female) at the University of Sydney providing 42,580 seconds of ground-truth data from laboratory and free-living activities (unpublished data). Laboratory activities were performed for 1.5 to 5 minutes each and included: 1) sitting and using a mobile phone or reading the paper; 2) standing and cleaning dishes; 3) walking on a treadmill (0% incline, 2.5% incline, and 7.5% incline); 4) walking in the corridor while carrying grocery bags in 1 hand; 5) walking in the corridor while carrying grocery bags in both hands , 5) running on a treadmill; and 6) using a stair-climber.

A subset of participants completed ambulatory free-living activities while being recorded by a researcher for up to 20 minutes with a hand-held Go-Pro, outside of the laboratory while engaging in any ambulatory activities they chose for any duration for each activity type. Video files were used to ascertain ground-truth physical activity.

Physical activity type and posture prediction results are shown below:

Binary decision tree classifier confusion matrix

		Prediction				
		**Sit**	**Stand[t]**	**Walk**	**Stairs**	**Run**
Ground-truth	**Sit**	**5,900 (100%)**	-	-	-	-
	**Stand**	-	**5,633 (95.5%)**	267 (4.5%)	-	-
	**Level walking**	-	189 (1.4%)	**11,894 (88.1%)**	103 (0.8%)	1,314 (9.7%)
	**Incline walking***	-	-	**6,962 (82.9)**	69 (0.8%)	1,369 (16.3%)
	**Stairs**	-	449 (7.3%)	786 (12.8%)	**4,534 (74.1%)**	351 (5.7%)
	**Run**	-	-	38 (1.4%)	47 (1.7%)	**2,675 (96.9%)**

Bold indicates correct predictions; *walking occurred on a treadmill at 2.5% and 7.5% incline.
[t]Includes standing still and standing utilitarian movements.

Binary decision tree classifier performance

	Sit	Stand	Walk	Stairs	Run	
Sensitivity	100	95.5	88.1	74.1	96.9	
Specificity	100	98.3	94.7	99.4	92.3	
F-score	100	92.6	90.1	83.4	63.2	
Negative predictive value	100	99.3	86.6	95.8	99.8	
Positive predictive value	100	89.8	94.5	95.4	46.9	
Overall accuracy						88.3%
Kappa statistic						0.84
Overall F-score						85.9%

PRISMA-IPD Checklist of items to include when reporting a systematic review and meta-analysis of individual participant data (IPD)

PRISMA-IPD Section/topic	Item No	Checklist item	Reported on page
**Title**			
Title	1	Identify the report as a systematic review and meta-analysis of individual participant data.	1
**Abstract**			
Structured summary	2	Provide a structured summary including as applicable:	4-5
		**Background**: state research question and main objectives, with information on participants, interventions, comparators and outcomes.	
		**Methods**: report eligibility criteria; data sources including dates of last bibliographic search or elicitation, noting that IPD were sought; methods of assessing risk of bias.	
		**Results**: provide number and type of studies and participants identified and number (%) obtained; summary effect estimates for main outcomes (benefits and harms) with confidence intervals and measures of statistical heterogeneity. Describe the direction and size of summary effects in terms meaningful to those who would put findings into practice.	
		**Discussion:** state main strengths and limitations of the evidence, general interpretation of the results and any important implications.	
		**Other:** report primary funding source, registration number and registry name for the systematic review and IPD meta-analysis.	
**Introduction**			
Rationale	3	Describe the rationale for the review in the context of what is already known.	7
Objectives	4	Provide an explicit statement of the questions being addressed with reference, as applicable, to participants, interventions, comparisons, outcomes and study design (PICOS). Include any hypotheses that relate to particular types of participant-level subgroups.	8
**Methods**			
Protocol and registration	5	Indicate if a protocol exists and where it can be accessed. If available, provide registration information including registration number and registry name. Provide publication details, if applicable.	NA
Eligibility criteria	6	Specify inclusion and exclusion criteria including those relating to participants, interventions, comparisons, outcomes, study design and characteristics (e.g. years when conducted, required minimum follow-up). Note whether these were applied at the study or individual level i.e. whether eligible participants were included (and ineligible participants excluded) from a study that included a wider population than specified by the review inclusion criteria. The rationale for criteria should be stated.	9

Identifying studies - information sources	7	Describe all methods of identifying published and unpublished studies including, as applicable: which bibliographic databases were searched with dates of coverage; details of any hand searching including of conference proceedings; use of study registers and agency or company databases; contact with the original research team and experts in the field; open adverts and surveys. Give the date of last search or elicitation.	9-11
Identifying studies - search	8	Present the full electronic search strategy for at least one database, including any limits used, such that it could be repeated.	9
Study selection processes	9	State the process for determining which studies were eligible for inclusion.	9
Data collection processes	10	Describe how IPD were requested, collected and managed, including any processes for querying and confirming data with investigators. If IPD were not sought from any eligible study, the reason for this should be stated (for each such study).<br><br>If applicable, describe how any studies for which IPD were not available were dealt with. This should include whether, how and what aggregate data were sought or extracted from study reports and publications (such as extracting data independently in duplicate) and any processes for obtaining and confirming these data with investigators.	9-11
Data items	11	Describe how the information and variables to be collected were chosen. List and define all study level and participant level data that were sought, including baseline and follow-up information. If applicable, describe methods of standardising or translating variables within the IPD datasets to ensure common scales or measurements across studies.	9-11, ESM Tables 1-2
IPD integrity	A1	Describe what aspects of IPD were subject to data checking (such as sequence generation, data consistency and completeness, baseline imbalance) and how this was done.	9-12, ESM Tables 1-2
Risk of bias assessment in individual studies.	12	Describe methods used to assess risk of bias in the individual studies and whether this was applied separately for each outcome. If applicable, describe how findings of IPD checking were used to inform the assessment. Report if and how risk of bias assessment was used in any data synthesis.	NA
Specification of outcomes and effect measures	13	State all treatment comparisons of interests. State all outcomes addressed and define them in detail. State whether they were pre-specified for the review and, if applicable, whether they were primary/main or secondary/additional outcomes. Give the principal measures of effect (such as risk ratio, hazard ratio, difference in means) used for each outcome.	12
Synthesis methods	14	Describe the meta-analysis methods used to synthesise IPD. Specify any statistical methods and models used. Issues should include (but are not restricted to):<br>• Use of a one-stage or two-stage approach.<br>• How effect estimates were generated separately within each study and combined across studies (where applicable).<br>• Specification of one-stage models (where applicable) including how clustering of patients within studies was accounted for.<br>• Use of fixed or random effects models and any other model assumptions, such as proportional hazards.<br>• How (summary) survival curves were generated (where applicable).	9-12, ESM Tables 1-2

		• Methods for quantifying statistical heterogeneity (such as I^2 and τ^2).<br>• How studies providing IPD and not providing IPD were analysed together (where applicable).<br>• How missing data within the IPD were dealt with (where applicable).	
Exploration of variation in effects	A2	If applicable, describe any methods used to explore variation in effects by study or participant level characteristics (such as estimation of interactions between effect and covariates). State all participant-level characteristics that were analysed as potential effect modifiers, and whether these were pre-specified.	12
Risk of bias across studies	15	Specify any assessment of risk of bias relating to the accumulated body of evidence, including any pertaining to not obtaining IPD for particular studies, outcomes or other variables.	NA
Additional analyses	16	Describe methods of any additional analyses, including sensitivity analyses. State which of these were pre-specified.	12-13
Results			
Study selection and IPD obtained	17	Give numbers of studies screened, assessed for eligibility, and included in the systematic review with reasons for exclusions at each stage. Indicate the number of studies and participants for which IPD were sought and for which IPD were obtained. For those studies where IPD were not available, give the numbers of studies and participants for which aggregate data were available. Report reasons for non-availability of IPD. Include a flow diagram.	Table 1, ESM Table 3
Study characteristics	18	For each study, present information on key study and participant characteristics (such as description of interventions, numbers of participants, demographic data, unavailability of outcomes, funding source, and if applicable duration of follow-up). Provide (main) citations for each study. Where applicable, also report similar study characteristics for any studies not providing IPD.	Table 1, ESM Table 3
IPD integrity	A3	Report any important issues identified in checking IPD or state that there were none.	ESM Tables 1-2
Risk of bias within studies	19	Present data on risk of bias assessments. If applicable, describe whether data checking led to the up-weighting or down-weighting of these assessments. Consider how any potential bias impacts on the robustness of meta-analysis conclusions.	NA
Results of individual studies	20	For each comparison and for each main outcome (benefit or harm), for each individual study report the number of eligible participants for which data were obtained and show simple summary data for each intervention group (including, where applicable, the number of events), effect estimates and confidence intervals. These may be tabulated or included on a forest plot.	Figures 1-3
Results of syntheses	21	Present summary effects for each meta-analysis undertaken, including confidence intervals and measures of statistical heterogeneity. State whether the analysis was pre-specified, and report the numbers of studies and participants and, where applicable, the number of events on which it is based.	13-17

		When exploring variation in effects due to patient or study characteristics, present summary interaction estimates for each characteristic examined, including confidence intervals and measures of statistical heterogeneity. State whether the analysis was pre-specified. State whether any interaction is consistent across trials.<br>Provide a description of the direction and size of effect in terms meaningful to those who would put findings into practice.	
Risk of bias across studies	22	Present results of any assessment of risk of bias relating to the accumulated body of evidence, including any pertaining to the availability and representativeness of available studies, outcomes or other variables.	NA
Additional analyses	23	Give results of any additional analyses (e.g. sensitivity analyses). If applicable, this should also include any analyses that incorporate aggregate data for studies that do not have IPD. If applicable, summarise the main meta-analysis results following the inclusion or exclusion of studies for which IPD were not available.	16-17
**Discussion**			
Summary of evidence	24	Summarise the main findings, including the strength of evidence for each main outcome.	17-20
Strengths and limitations	25	Discuss any important strengths and limitations of the evidence including the benefits of access to IPD and any limitations arising from IPD that were not available.	21-22
Conclusions	26	Provide a general interpretation of the findings in the context of other evidence.	22-23
Implications	A4	Consider relevance to key groups (such as policy makers, service providers and service users). Consider implications for future research.	17-20
**Funding**			
Funding	27	Describe sources of funding and other support (such as supply of IPD), and the role in the systematic review of those providing such support.	23-24
